# Fasting appetite-related gut hormone responses after weight loss induced by calorie restriction, exercise, or both in people with overweight or obesity: a meta‐analysis

**DOI:** 10.1038/s41366-025-01726-4

**Published:** 2025-02-10

**Authors:** Zhuoxiu Jin, Jiajin Li, Alice E. Thackray, Tonghui Shen, Kevin Deighton, James A. King, David J. Stensel

**Affiliations:** 1 National Centre for Sport and Exercise Medicine, School of Sport, Exercise and Health Sciences, Loughborough University, Loughborough, United Kingdom; 2 National Institute for Health and Care Research (NIHR) Leicester Biomedical Research Centre, University Hospitals of Leicester National Health Service (NHS) Trust and the University of Leicester, Leicester, United Kingdom; 3 Department of Physical Education and Aesthetic Education, Hangzhou City University, Hangzhou, China; 4 Nuffield Health, Epsom, Surrey, United Kingdom; 5 Faculty of Sport Sciences, Waseda University, Tokorozawa, Japan; 6 Department of Sports Science and Physical Education, The Chinese University of Hong Kong, Ma Liu Shui, Hong Kong

**Keywords:** Obesity, Homeostasis

## Abstract

**Objectives:**

Altered appetite-related gut hormone concentrations may reflect a physiological adaptation facilitating weight regain after weight loss. This review investigates hormonal changes after weight loss achieved through calorie restriction (CR), exercise (EX), or both combined (CREX).

**Methods:**

A systematic search of PubMed (MEDLINE), EMBASE, SPORTDiscus, Cochrane Library, Web of Science, and ClinicalTrials.gov was conducted to identify randomised controlled trials (RCTs) and non-RCTs reporting in a fasting state either pre- and post-intervention appetite-related hormone concentrations or the changes therein after weight loss. The hormones examined were ghrelin, peptide tyrosine tyrosine (PYY), glucagon-like peptide -1 (GLP-1), and cholecystokinin (CCK), in their total and/or active form. Standardised mean differences (SMD) were extracted as the effect size.

**Results:**

127 studies were identified: 19 RCTs, 108 non-RCTs, 1305 and 4725 participants, respectively. In response to weight loss induced by CR, EX or CREX, the meta-analysis revealed an increase in total ghrelin from both RCTs (SMD: 0.55, 95% CI: 0.07–1.04) and non-RCTs (SMD: 0.24, 95% CI: 0.14–0.35). A decrease in acylated ghrelin was identified for RCTs (SMD: –0.58, 95% CI: –1.09 to –0.06) but an increase was observed for non-RCTs (SMD: 0.15, 95% CI: 0.03 to 0.27). Findings also revealed a decrease in PYY (total PYY: SMD: –0.17, 95%CI: –0.28 to –0.06; PYY_3-36_: SMD: –0.17, 95%CI: –0.32 to –0.02) and active GLP-1 (SMD: -0.16, 95% CI: –0.28 to –0.05) from non-RCTs. Changes in hormones did not differ among the three interventions when controlling for weight loss. Meta-regression indicated that greater weight loss was associated with a greater increase in total ghrelin.

**Conclusions:**

Weight loss induced by CR, EX, or CREX elicits an increase in total ghrelin, but varied responses in other appetite-related hormones. The extent of weight loss influences changes in appetite-related gut hormone concentrations.

## Introduction

Obesity is a major public health concern with the prevalence remaining high globally, bringing healthcare challenges across countries [1, 2]. Among people who are successful at losing weight through diet alone or diet combined with exercise, individuals regain, on average, 80% of the lost weight within 5 years [3]. The reasons for weight regain after weight loss are multifactorial and include compensatory physiological mechanisms aimed at resisting weight loss and promoting weight regain [4].

Appetite related gut hormones including ghrelin, peptide tyrosine tyrosine (PYY), glucagon-like peptide 1 (GLP-1), and cholecystokinin (CCK), have strong influences on appetite and energy intake. Ghrelin, functions as an orexigenic hormone, stimulating appetite by acting on the hypothalamus [5]. In contrast, the anorexigenic hormones PYY, GLP-1, and CCK promote postprandial satiation and satiety by slowing gastric emptying and transmitting signals to the hypothalamus in proportion to caloric intake [6]. Exogenous infusion of these appetite hormones may increase/decrease subjective appetite feelings and energy intake [7–10]. In the long term, dietary induced weight loss is accompanied by reduced concentrations of total PYY and CCK and increased concentrations of acylated ghrelin and these changes have been shown to precede weight regain in people with obesity [11]. However, it is important to note that a direct association between these hormonal changes and weight regain remains inconclusive, as some studies have reported an uncoupling effect where hormone fluctuations do not consistently align with changes in appetite or food intake [12–14].

Calorie restriction (CR) and exercise (EX) are two primary interventions for weight management via reducing energy intake and increasing energy expenditure, respectively. One study has demonstrated that acute periods of energy restriction via diet elicit compensatory changes in appetite perceptions and appetite related hormones favouring increases in energy intake that were not observed after acute increases in energy expenditure via exercise [15]. This may be because elevated interleukin-6 (IL-6) concentrations in response to exercise stimulate PYY and GLP-1 levels [16] and suppress ghrelin expression [17], along with a redistribution of blood flow from gut hormone release sites to active muscles that may decrease acylated ghrelin due to decreased activity of the ghrelin O-acyl transferase (GOAT) enzyme, which is required to acylate ghrelin into its active form [18]. In the long-term, chronic CR has been shown to enhance perceived and physiological urges to eat [19, 20]. Conversely, habitual exercise appears to exert a dual effect on subjective appetite sensations and appetite-related hormones by increasing motivation to eat in a fasted state but enhancing postprandial satiety [21, 22]. This suggests that chronic exercise may influence appetite control differently than calorie restriction. The mechanism is unclear, but differences in appetite-related hormone changes to these interventions may be involved.

Two systematic reviews have assessed changes in circulating appetite-related hormones after dietary induced weight loss, which identified a decrease in PYY, GLP-1, and CCK, alongside an increase in ghrelin concentrations, in their total or active form [23, 24]. A meta-analysis reported no statistically significant changes in circulating total ghrelin, acylated ghrelin, and total PYY or PYY_3-36_ after exercise-induced weight loss in adults with overweight and obesity [25]. However, the small number of studies analysed for each hormone leaves gaps in understanding. Furthermore, no review has directly compared responses between exercise and diet induced weight loss. The current meta-analysis therefore included both randomised controlled trials (RCTs) and non-RCTs to reduce the gap in understanding and address the differences among interventions.

Additional factors may influence appetite hormone changes after weight loss. Research suggests that fat-free mass (FFM) is a major driver of energy intake compared to fat mass (FM), particularly under conditions of energy balance and negative energy balance [26]. While the exact mechanisms through which FFM modulates appetite are not fully understood, it has been proposed that myokines released during FFM loss may act at the brain level, potentially impacting circulating concentrations of appetite-related hormones [27]. Furthermore, ghrelin, which is associated with muscle growth, has been implicated in myocyte proliferation [28]. FFM loss after energy restriction may activate the preproghrelin gene, thereby stimulating ghrelin production and promoting myoblast differentiation [29]. Additionally, women tend to exhibit greater compensatory responses to CR, likely driven by hormone-mediated energy conservation mechanisms aimed at preserving fat stores for reproductive function [30]. The rate of weight loss also impacts body composition changes: while no significant differences in FFM loss have been observed between rapid and gradual weight loss, gradual weight loss is associated with a greater reduction in FM, resulting in a higher FM-to-FFM loss ratio [31].

The primary aim was to investigate the effects of weight loss achieved via CR, EX, or a combination of both (CREX) on the circulating concentrations of ghrelin, PYY, GLP-1, and CCK in their total or active forms, and to compare responses among these three interventions. The secondary aim was to identify potential contributors, specifically FFM loss, sex, and rate of weight loss, to hormonal responses after dietary and exercise-induced weight loss as well as to examine the difference between RCTs and non-RCTs. By addressing these aims, this meta-analysis provides a comprehensive overview of appetite-related gut hormone responses to weight loss.

## Methods

This systematic review with meta-analysis was conducted according to the Preferred Reporting Items for Systematic Reviews and Meta-analyses (PRISMA) guidelines [32] and was registered prospectively on the PROSPERO database (CRD42022295939).

### Search strategy

The following databases were searched for studies in humans published in the English language: PubMed (MEDLINE), EMBASE, SPORTDiscus, Cochrane Library, Web of Science and ClinicalTrials.gov, with no restriction placed on the date of publication (from inception to March 2022), including published abstracts. The reference list of all selected articles and relevant reviews was manually screened to identify additional studies not captured in the initial search. The searches were re-run before the final analysis to identify any further studies that met the inclusion criteria (15th of March 2024). We used search terms related to weight loss, CR or EX interventions, and gut hormone concentrations, with MeSH terms utilised where appropriate (details of the specific search strategy for each database can be found in Supplementary Information Content 1).

### Inclusion and exclusion criteria

The following criteria were applied to identify articles for inclusion in this systematic review and meta-analysis:

- RCTs or non-RCTs.
- Studies were included if statistically significant weight loss was achieved and at least one of the relevant appetite hormones in total or active form (ghrelin/PYY/GLP-1/CCK) was measured in an overnight fasting state.
- Participants were adults >18 years of age, with overweight or obesity (BMI ≥ 25 kg/m^2^). Studies were excluded if the participants were pregnant, had illnesses including diabetes, active infectious disease, severe cardiovascular disease and diseases that require medications affecting body weight or hormone replacement therapy.

- Studies were included if CR, EX or CREX was one of the interventions. Any dietary intervention that achieved CR, such as the ketogenic diet, Mediterranean diet, or palaeolithic diet, was also included. However, studies were not included if participants completed other interventions together with diet or exercise, such as bariatric surgery or pharmacological treatment.

### Selection process

The articles identified were electronically imported into an online software platform (Covidence, Veritas Health Innovation, Melbourne, Australia) for screening and duplicate studies were automatically or manually removed. Three reviewers (ZJ, AT & TS) independently screened the titles and abstracts of retrieved articles to evaluate their eligibility for inclusion in the review and later compared notes to reach a consensus. Potentially relevant articles were sourced, and the full-text articles were screened against the criteria above (ZJ & TS). Any disagreements in study selection were discussed and resolved by discussion or by consulting a third investigator (AT).

### Data extraction

Data were extracted by two reviewers (ZJ & JL) independently. Any disagreements were resolved by discussion until consensus was reached or by consulting a third investigator (AT). The following information was extracted: (1) study information (author(s), publication date); (2) study design; (3) sample size (distribution per study arm, attrition); (4) participant characteristics (age, sex, body weight, body mass index, comorbidities, medication use); (5) details of the intervention(s); (6) details of the control arm if RCTs; and (7) appetite-related hormone concentrations (mean pre-intervention, post-intervention and absolute change in concentration with a measure of variance (SD, SEM or 95% CI)). For the studies including assessments at multiple time-points, the data at baseline and immediately after the weight loss phase were extracted for the analysis.

### Risk of bias (quality) assessment

Two reviewers (ZJ & JL) independently assessed the quality of the selected studies for risk of bias. Cochrane’s risk-of-bias tool 2.0 [33] was used for assessing RCTs. Items were evaluated in three categories: low risk of bias, some concerns and high risk of bias. The ROBINS-I tool [34] was used to assess risk of bias for non-RCTs. Studies were classified as (1) low risk of bias (2) moderate risk of bias (3) serious risk of bias; (4) critical risk of bias; and (5) no information.

### Statistical analysis

A random-effects meta-analysis was conducted using the Metafor package [35] in R version 4.2.2 [36]. This meta-analysis was performed for each appetite-related hormone separately, and the primary analysis focused on the changes of each hormone after weight loss induced by any intervention. Sample size, mean of the changes and SD of the changes for intervention and control (RCTs only) groups were extracted and used in the analyses. In circumstances in which only pre- and post-intervention means and SDs were reported, the correlation coefficient was used to calculate the SD of the changes. Correlations were determined using standard methods of back-calculation from the included studies that provided pre- and post- intervention mean, SD, and SD of the changes [37]. The mean of these correlation coefficients was used in the calculation of relevant effect sizes. Sensitivity analyses were performed incorporating a wide range of correlation coefficients, starting from 0.1 to 0.9 in increments of 0.1. In addition, the sample size, means and the exact p-value of the difference were used from studies where SDs were not available, according to standard methods [37]. Standard error of the mean and confidence intervals (CI) were back calculated to SD, as required, using standard methods [37]. Median and IQR were also back transformed using standard methods [38]. For instances where data was not explicitly reported, we contacted the corresponding authors to request access to the raw data. In cases where data was presented in graphical format within figures, an online tool (WebPlotDigitizer, https://apps.automeris.io/wpd/) was used to extract the numerical values. Standardised mean difference (SMD) was used as the effect size. The effect size was calculated using multilevel modelling to account for the inherent dependency between multiple independent groups originating from the same studies. A minimum of at least five intervention groups was required to perform a meta-analysis of respective variables.

Subgroup analyses were undertaken to investigate the influence of intervention type (CR vs. EX vs. CREX) and study design (RCT vs. non-RCT) for all included studies. In non-RCTs, sensitivity analyses were conducted using a published method [39–41] to evaluate the potential influence of unmeasured confounding and other sources of bias. E-values were calculated to estimate the minimum strength of association that an unmeasured confounder would require with both the intervention and the outcome to fully account for the observed associations. Meta-regression analysis was performed to investigate the relationship between changes in appetite hormone concentrations in response to the extent of weight loss (%). The degree of weight change was calculated as follows: formula A (RCTs) and formula B (non-RCTs) were applied for studies reporting the mean of weight changes, and formula C (RCTs) and formula D (non-RCTs) were applied for studies reporting body weight at pre- and post-intervention timepoints:

A. ((Mean of the changes_int_/Pre-weight_int_) – (Mean of the changes_con_/Pre-weight_con_)) * 100
B. Mean of the changes_int_/Pre-weight_int_ * 100
C. (((Pre-weight_int_ – Post-weight_int_)/Pre-weight_int_) – ((Pre-weight_con_ – Post-weight_con_)/ Pre-weight_con_))) * 100
D. ((Pre-weight_int_ – Post-weight_int_)/Pre-weight_int_) * 100

Subscripts ‘int’ and ‘con’ here mean intervention group and control group. The same calculations were applied to the extent of FFM loss. Exploratory analyses were undertaken to investigate whether the magnitude of FFM loss alone acts as a potential modifier. Additional analyses explored (1) the role of sex on gut hormone changes while controlling for the magnitude of weight loss and (2) the role of the rate of weight loss on gut hormone changes by examining study duration while controlling for the total amount of weight lost. Heterogeneity between trials was estimated using restricted maximum likelihood and assessed using the Chi-squared statistic (Q value) and the I-squared statistic. The Cochrane Handbook offers a rough guide for the interpretation of the I-squared statistic. An I^2^ value ranging from 0 to 40% may not represent significant heterogeneity. Moderate heterogeneity is indicated by an I^2^ value between 30 and 60%. Values ranging from 50 to 90% point towards substantial heterogeneity. Lastly, an I^2^ value of 75% to 100% is indicative of considerable heterogeneity [42]. Case-deletion diagnostics were performed to investigate influential studies within each analysis. This included the determination of externally studentised residuals, dffits (difference in fits) values, Cook’s distances, covariance ratios, estimates of τ^2^, estimates of the Q-statistic, hat values, weight and dfbetas (difference in fits for beta estimates) values, when each study was removed in turn. Sensitivity analyses were conducted through the repetition of main effect analyses after the exclusion of influential studies, to determine whether these studies markedly affected the overall conclusions. Findings are presented with the inclusion of influential studies if statistical significance was unaffected by their inclusion. Small study effects were explored with funnel plots and Egger’s test. All the analysis and plots were performed using RStudio version 2023.03.0 + 386 running R version 4.2.2 (RStudio, PBC, Boston, MA). *P* < 0.05 was set as statistical significance.

## Results

### Search results

**Fig. 1:**
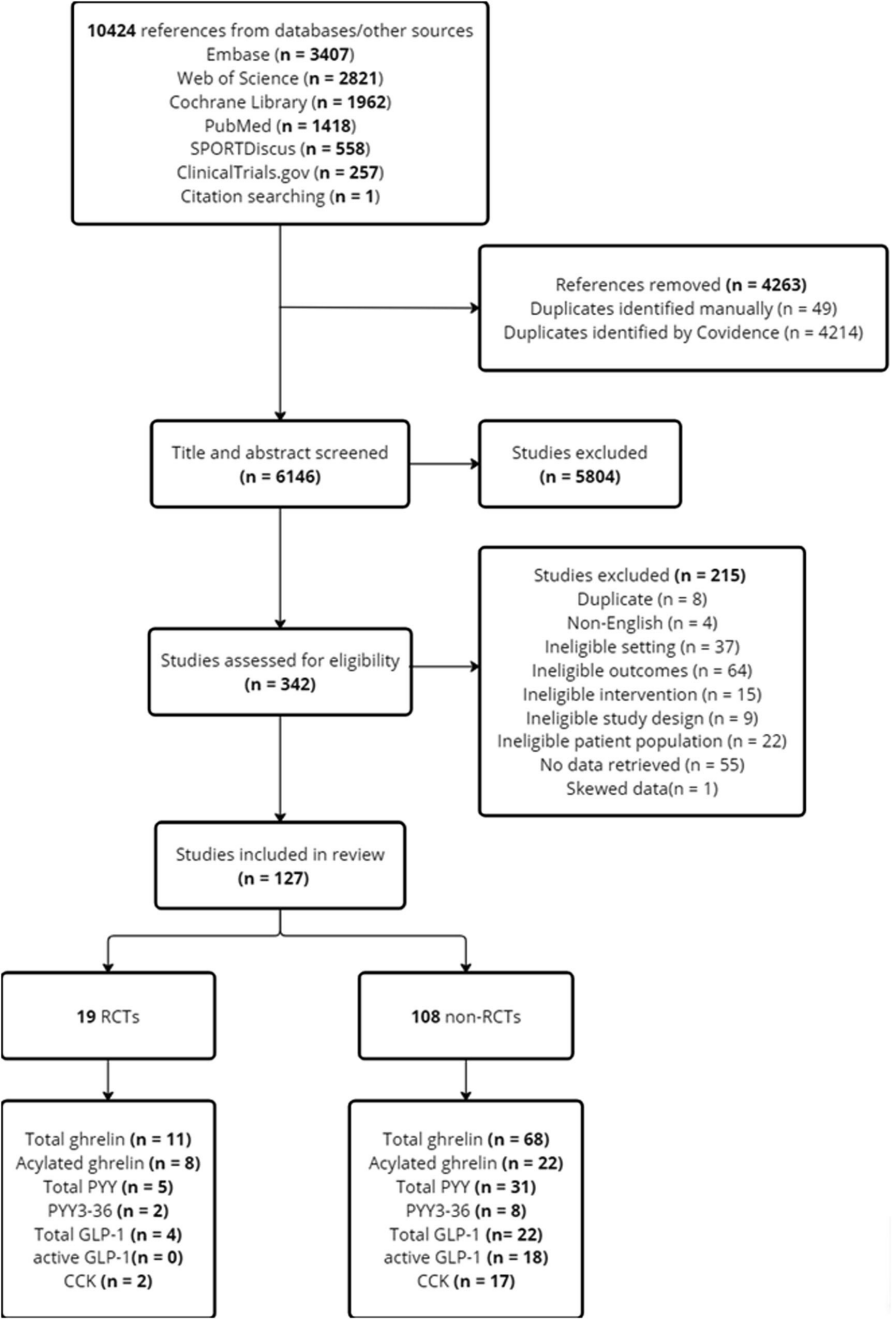
Flow diagram of the selection process.

The systematic review initially included 128 studies. However, one study [43] was excluded from the meta-analysis due to its exceptionally large effect sizes, which were between ten and twenty times higher than those observed in the other studies and significantly skewed the results according to the sensitivity analyses. Therefore, a total of 127 studies were included, of which 19 studies were RCTs [44–62] involving 1305 participants, and 108 studies were non-RCTs [11, 63–169] involving 4725 participants. The selection flow diagram is shown in Fig. 1.

Participants ranged in age from 18 to 70 years, with baseline BMI requirements varying from 25 to 50 kg/m². The weighted average duration of the studies was 20 weeks (ranging from 4 to 52 weeks) for RCTs, and 16 weeks (ranging from 0.5 to 104 weeks) for non-RCTs. On average, RCTs reported a 6% weight loss, while non-RCTs reported a 7% weight loss. CR interventions reported an average weight loss of 9% in RCTs and 7% in non-RCTs. EX interventions elicited a 4% weight loss in RCTs and 3% in non-RCTs. CREX interventions demonstrated a 13% weight loss in RCTs and an 8% weight loss in non-RCTs.

The CR interventions employed an array of approaches, ranging from straightforward daily calorie reduction to more intricate methods. For RCTs, one study applied simple calorie restriction, while three studies combined calorie restriction with macronutrient variations (1 focused on protein maintenance and 2 on restricted fat intake). Additionally, one study utilized intermittent fasting. Among the non-RCTs, 56 studies applied straightforward calorie restriction, with degrees of restriction ranging from very low-calorie diets (550-660 kcal/day) to approximately 1500 kcal/day or achieving a 1000 kcal deficit or 30%-40% of daily energy requirements. Nine studies incorporated macronutrient variations (low carb, low fat, or high protein). Eight studies included food supplements, such as increased dairy product intake to boost Ca^2+^, fibre from food, cholesterol through eggs, Brazil nuts, and low-fat yogurt. Seven studies used specific diets to achieve calorie restriction, including the Mediterranean diet, vegetarian diet, high or low satiating food, low energy density diet, low glycaemic index diet, fast-mimicking diet, paleo diet, and neurodegenerative delay diet. Four studies altered meal patterns, such as having a heavy breakfast or dinner, consuming carbohydrates at dinner, and varying meal frequency (e.g., 3 or 6 meals per day). Three studies employed intermittent fasting, allowing 0%-25% of energy requirements on fasting days.

The EX interventions included both aerobic training and resistance training. For RCTs, seven studies applied moderate-intensity continuous training (MICT), three studies applied high-intensity interval training (HIIT), and one study combined MICT and HIIT. Four studies focused on resistance training, with one study combining MICT and resistance training, and another combining resistance training and walking. Among non-RCTs, nine studies applied MICT, three applied HIIT, one allowed participants to choose between HIIT or MICT, two combined MICT and HIIT, one focused on resistance training, and one combined MICT with resistance training.

The CREX interventions included a wide array of protocols. For RCTs, one study applied a lifestyle intervention involving telemonitored lifestyle-induced weight loss programmes, and two studies combined CR with MICT. Among non-RCTs, seven studies applied lifestyle interventions aimed at increasing physical activity levels and decreasing energy intake, four studies combined CR with brisk walking, six studies combined CR with MICT (one incorporating a ketogenic diet), two studies combined CR with HIIT, and two studies combined CR with resistance training. Additionally, one study offered a choice between CR with resistance training or MICT (detailed study information is provided in Supplementary Table 1).

**Fig. 2:**
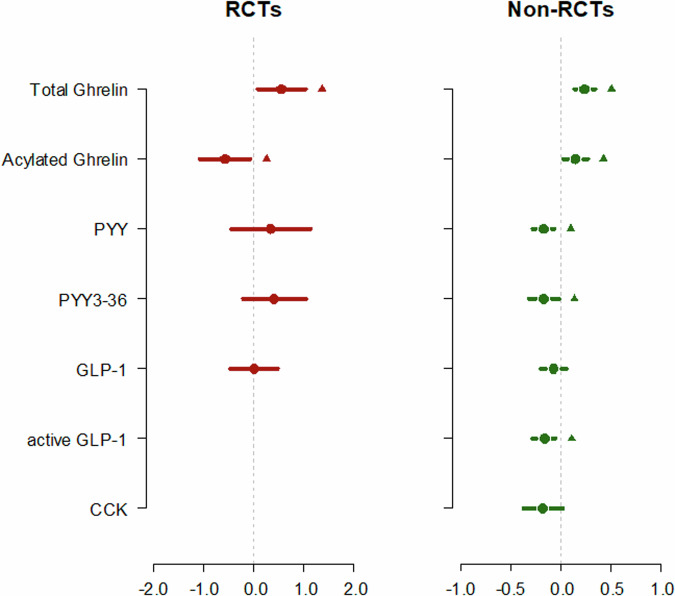
Pooled results from each meta-analysis for each outcome. The dots indicate the overall effect size, and the lines represent the 95% confidence interval. The triangle symbol indicates *p* < 0.05.

Sensitivity analyses across various correlation coefficients demonstrated consistent results (Supplementary Information Content 2). The results discussed below are based on the mean correlation coefficient values obtained from the included studies for each hormone under investigation. The primary analysis aimed to investigate the effects of weight loss achieved via CR, EX, or CREX on circulating appetite-related gut hormone concentrations. Separate meta-analyses were conducted to assess the effects of specific protocols within each intervention type on appetite-related hormones. However, due to a limited number of studies for some protocols, separate analyses were only feasible for examining the effects of MICT on total ghrelin in both RCTs and non-RCTs, as well as on acylated ghrelin in RCTs, and for CR interventions across all hormones in non-RCTs. The results were consistent regardless of whether protocols were analysed individually or grouped by intervention type. Therefore, all protocols within each intervention type were included in the final analysis. A summary graph of these pooled findings, incorporating each intervention type, is presented in Fig. 2.

### Circulating total ghrelin responses in RCTs

**Fig. 3:**
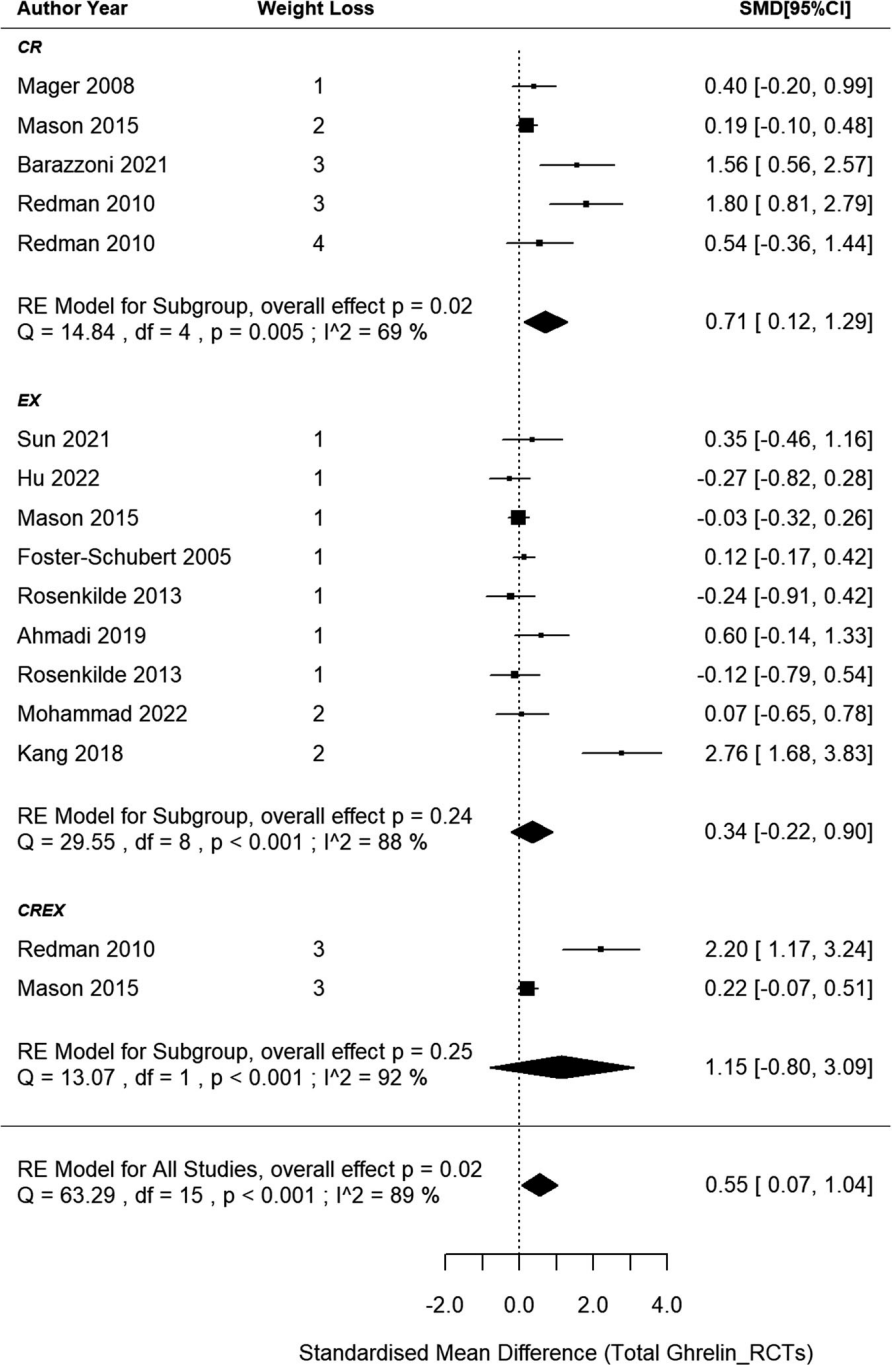
Total ghrelin changes after weight loss from RCTs. *N* = 11 studies with 16 intervention groups (5 CR, 9 EX, 2 CREX). RE model: random effects model. The weight loss column is defined as follows.1: less than 5% weight loss; 2: 5–10% weight loss, 3: 10–15% weight loss, and 4: 15–20% weight loss. The size of the squares is proportional to the weight of the study in the meta-analysis.

11 RCTs [44, 48, 49, 51, 55–59, 61, 62] were included in the meta-analysis, comprising 5 CR, 9 EX and 2 CREX independent intervention groups, and involving a total of 895 participants (Fig. 3). Findings revealed a pooled SMD of 0.55 (95% CI: 0.07–1.04; *p* = 0.024) in total ghrelin changes after weight loss with a high level of between-study heterogeneity across studies (I^2^ = 89%; *p* < 0.001). Meta-regression analysis identified that each 1% increase in weight loss was associated with a 0.09 (95% CI: 0.01–0.17; *p* = 0.020) unit increase in the SMD of total ghrelin, which explained 29% of the heterogeneity. The asymmetry in the funnel plot and the Egger’s test result suggests the possibility of publication bias (z = 3.89, *p* < 0.001, Supplementary Information Content 3 (SI3) - Fig. S1).

### Circulating total ghrelin responses in non-RCTs

**Fig. 4:**
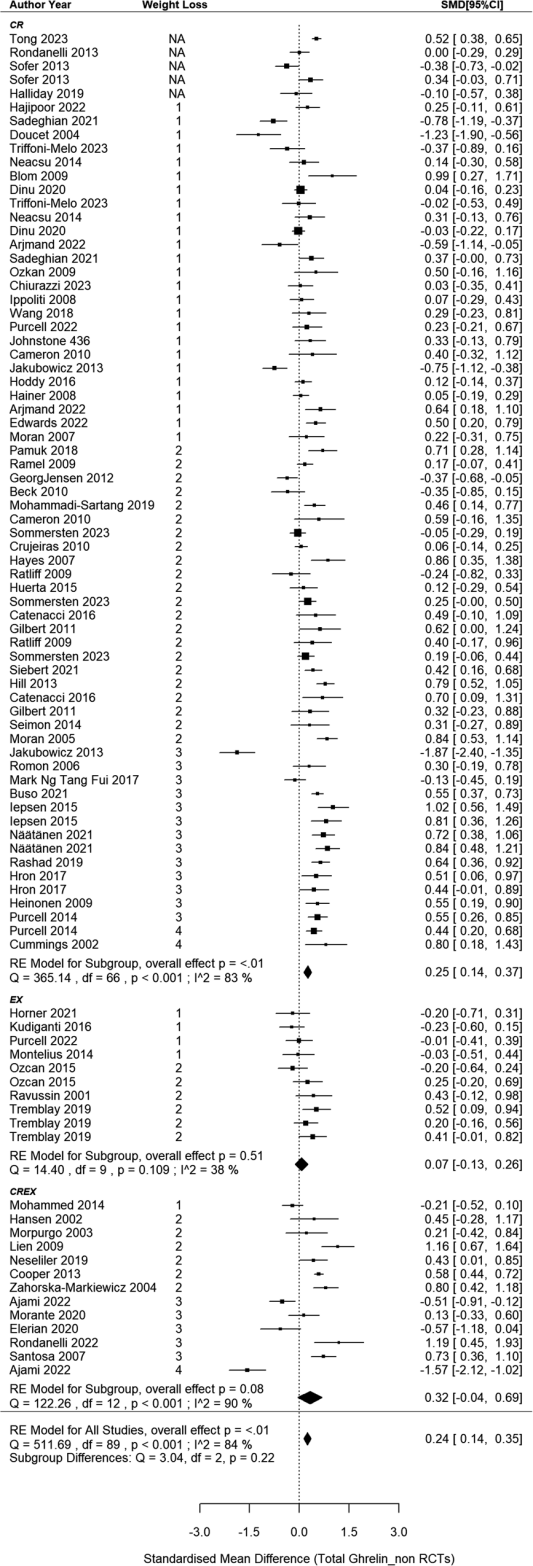
Total ghrelin changes after weight loss from non-RCTs. *N* = 68 studies with 90 intervention groups (67 CR, 10 EX and 13 CREX). RE model: random effects model. The weight loss column is defined as follows.1: less than 5% weight loss; 2: 5–10% weight loss, 3: 10–15% weight loss, and 4: 15–20% weight loss. NA indicates that studies reported significant weight loss without providing exact numbers. The size of the squares in the figure is proportional to the weight of each study in the meta-analysis.

The meta-analysis synthesized data from 68 non-RCTs [66, 68, 71, 72, 77–79, 81–85, 87–90, 102–110, 112–120, 122–141, 148–152, 157–159, 162–167], incorporating findings from 67 CR, 10 EX and 13 CREX intervention groups, with a cumulative sample size of 3194 participants (Fig. 4). A small-to-moderate pooled SMD of 0.24 (95% CI: 0.14–0.35; *p* < 0.001) was found, with considerable heterogeneity between studies (I^2^ = 84%; *p* < 0.001). Meta-regression revealed that each 1% increase in weight loss was associated with a significant increase of 0.03 SMD for total ghrelin (95% CI: 0.01–0.06; *p* = 0.013). Egger’s test showed no evidence of publication bias (z = –0.40, *p* = 0.691) which is supported by the funnel plot (SI3 - Fig. S2).

### Circulating acylated ghrelin responses in RCTs

**Fig. 5:**
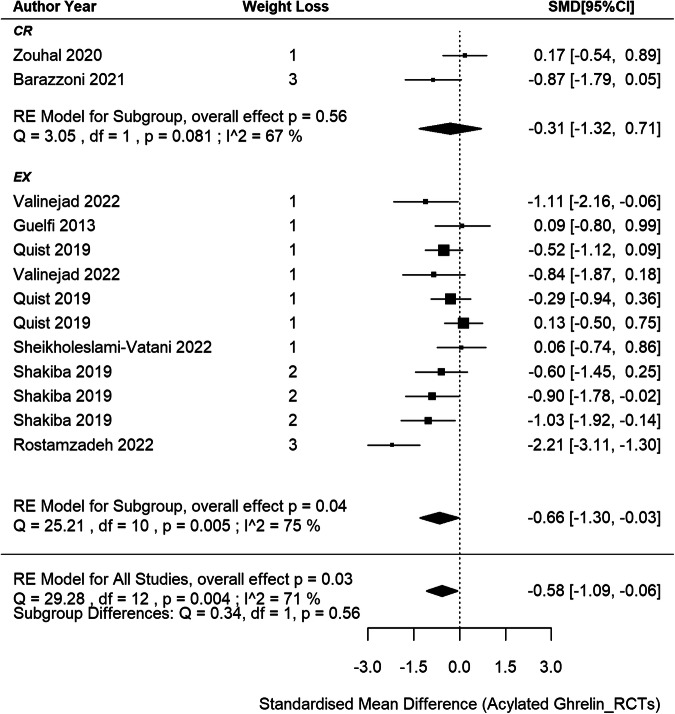
Acylated ghrelin changes after weight loss from RCTs. *N* = 8 studies and 13 intervention groups (2 CR and 11 EX). RE model: random effects model. The weight loss column is defined as follows.1: less than 5% weight loss; 2: 5–10% weight loss and 3: 10–15% weight loss. The size of the squares is proportional to the weight of the study in the meta-analysis.

8 RCTs [44, 46, 47, 50, 52–54, 60] were included in the meta-analysis, comprising 13 independent intervention groups (2 CR and 11 EX only, no CREX group), and involving a total of 288 participants (Fig. 5). Findings revealed a pooled SMD of –0.58 in acylated ghrelin concentration changes after weight loss (95% CI: –1.09 to –0.06; *p* = 0.028) with substantial between-studies heterogeneity (I^2^ = 71%, *p* = 0.004). Meta-regression analyses revealed each 1% increase in weight loss was associated with a 0.14 SMD decrease in acylated ghrelin (95% CI: –0.23 to –0.05, *p* = 0.004). This finding accounted for 67% of the observed heterogeneity. Furthermore, when this association was considered, the between-study heterogeneity notably decreased and became non-significant (I^2^ = 33%, *p* = 0.105). The asymmetry observed in the funnel plot (SI3 - Fig. S3) suggests a potential of publication bias, which is also indicated by the result in the Egger’s test (z = –2.22, *p* = 0.027).

### Circulating acylated ghrelin responses in non-RCTs

**Fig. 6:**
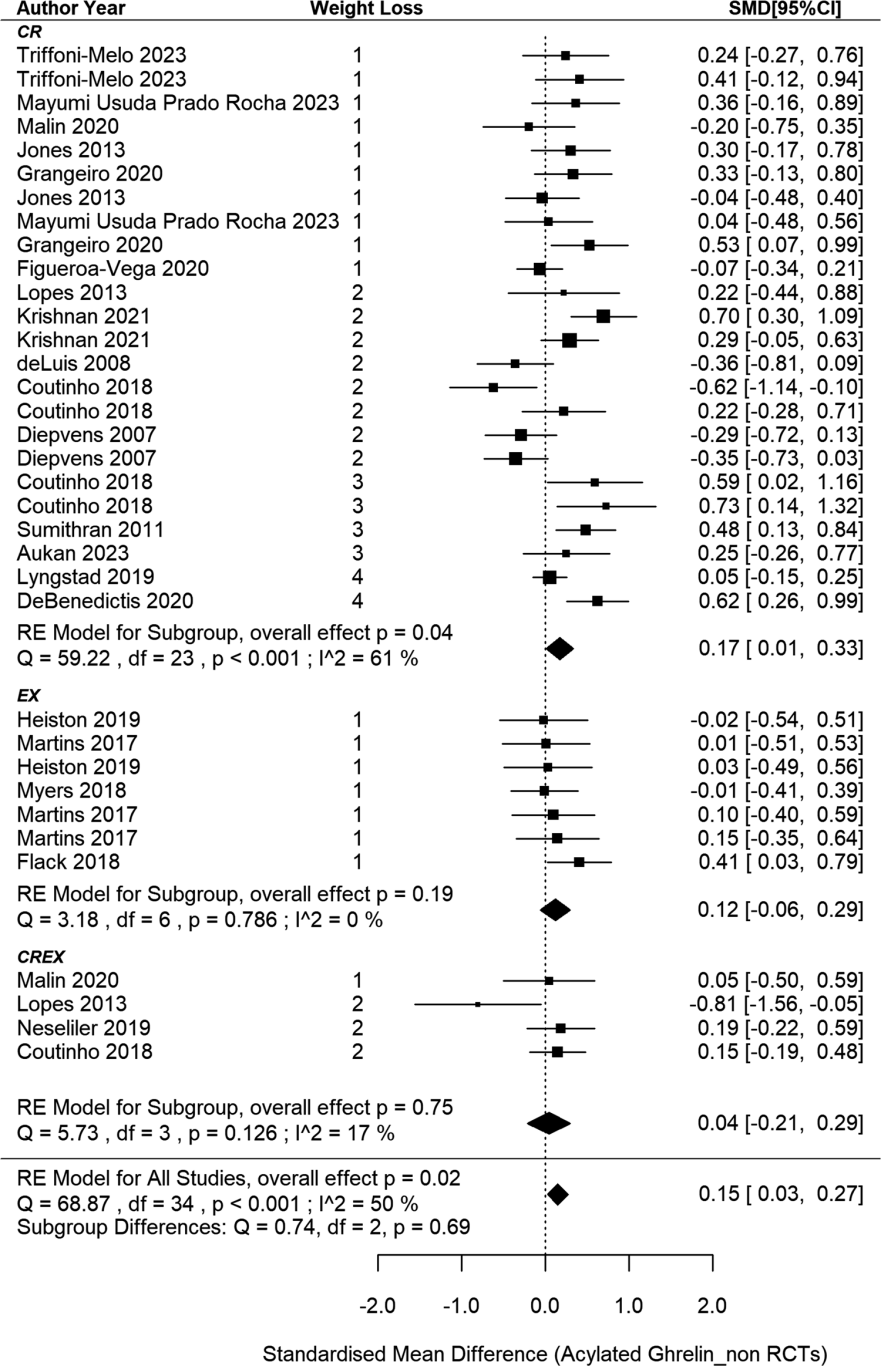
Acylated ghrelin changes after weight loss from non-RCTs. *N* = 22 studies and 35 intervention groups (24 CR, 7 EX and 4 CREX). RE model: random effects model. The weight loss column is defined as follows.1: less than 5% weight loss; 2: 5–10% weight loss, 3: 10–15% weight loss, and 4: 15–20% weight loss. NA indicates that studies reported significant weight loss without providing exact numbers. The size of the squares in the figure is proportional to the weight of each study in the meta-analysis.

This meta-analysis included 22 non-RCTs [11, 69, 70, 73, 75, 76, 80, 86, 91, 93, 95, 97, 98, 110, 142–145, 153, 156, 157, 161], aggregating data from 35 independent intervention groups (24 CR, 7 EX and 4 CREX) and comprising 793 participants (Fig. 6). A pooled SMD of 0.15 (95% CI: 0.03 to 0.27; *p* = 0.015) in acylated ghrelin changes was identified (I^2^ = 50%; *p* < 0.001). Meta-regression analysis did not reveal a significant association between weight loss magnitude and acylated ghrelin changes (*p* = 0.368). The funnel plot appeared to be symmetrical (SI3 - Fig. S4), suggesting a lack of publication bias. This observation is supported by the Egger’s test (z = –0.58; *p* = 0.562).

### Circulating total PYY responses in RCTs

**Fig. 7:**
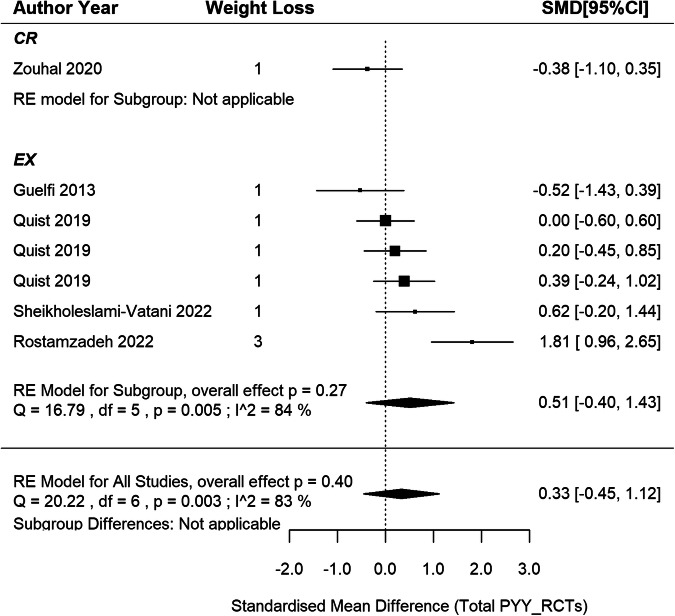
Total PYY changes after weight loss from RCTs. *N* = 5 studies and 7 intervention groups (1 CR and 6 EX). RE model: random effects model. The weight loss column is defined as follows.1: less than 5% weight loss; 2: 5–10% weight loss and 3: 10–15% weight loss. The size of the squares is proportional to the weight of the study in the meta-analysis.

5 RCTs [46, 47, 50, 53, 54] were included in the meta-analysis, comprising 6 EX groups and 1 CR group across the studies, involving 200 participants (Fig. 7). The meta-analysis revealed a non-significant pooled SMD of 0.33 in total PYY concentrations after weight loss (95% CI: –0.45 to 1.12; *p* = 0.404) with a substantial heterogeneity (I^2^ = 83%, *p* < 0.001). According to meta-regression analysis, a significant positive association was observed between the extent of weight loss and the corresponding changes in PYY concentrations, showing that each 1% increase in weight loss led to a 0.21 SMD increase in PYY (95% CI: 0.11–0.31; *p* < 0.001), which accounts for 100% of the observed heterogeneity. Similar results were obtained when the analysis included EX interventions only (slope: 0.20, 95%CI: 0.10–0.30, *p* < 0.001, accounting for 100% of the observed heterogeneity). The symmetry in the funnel plot and the Egger test indicates no publication bias (z = 0.49, *p* = 0.628; SI3 - Fig. S5).

### Circulating total PYY responses in non-RCTs

**Fig. 8:**
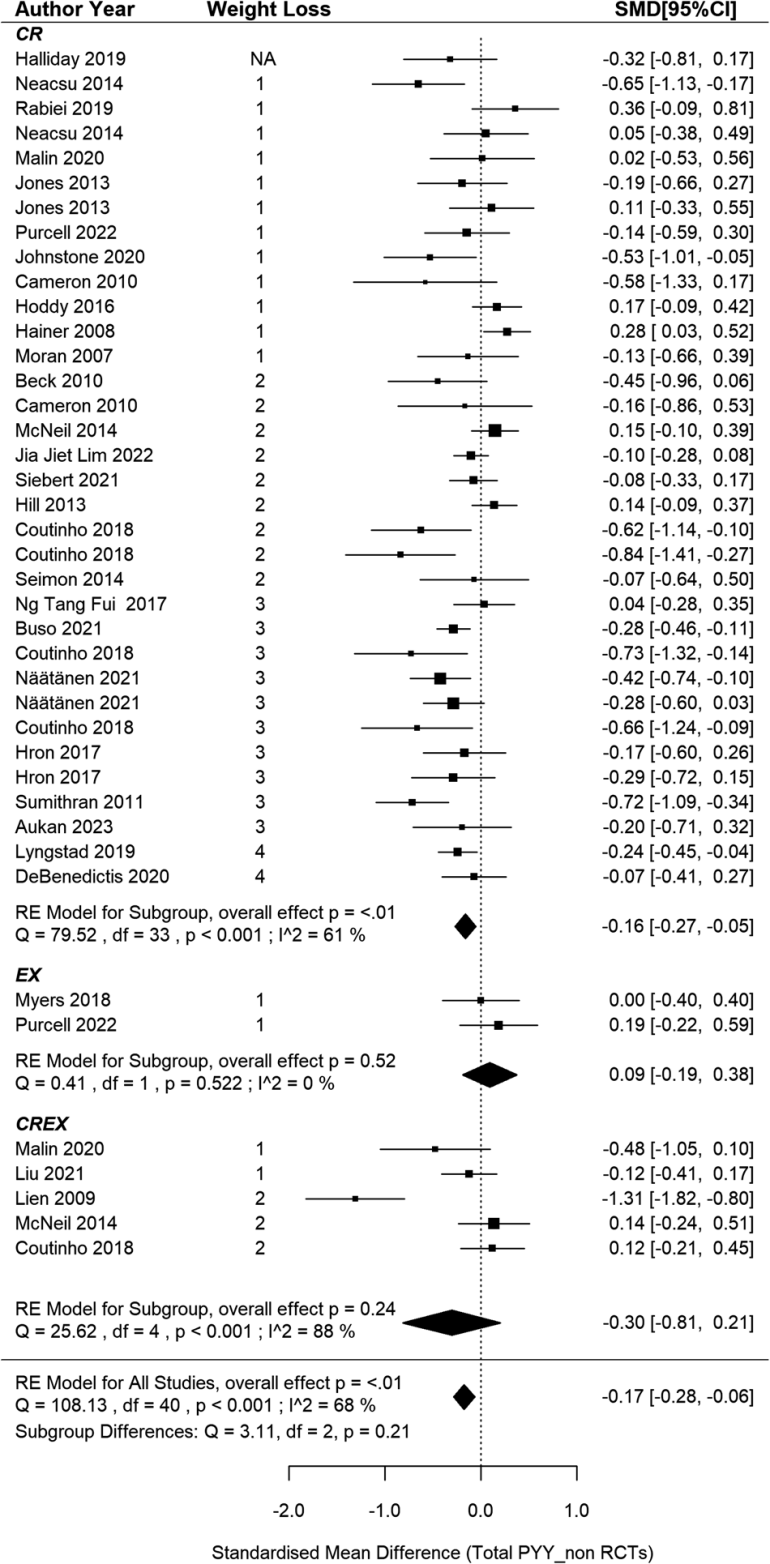
Total PYY changes after weight loss from non-RCTs. *N* = 31 studies and 41 intervention groups (34 CR, 2EX and 5 CREX). RE model: random effects model. The weight loss column is defined as follows.1: less than 5% weight loss; 2: 5–10% weight loss, 3: 10–15% weight loss, and 4: 15–20% weight loss. The size of the squares is proportional to the weight of the study in the meta-analysis.

This meta-analysis included 31 non-randomised studies [11, 66, 69, 70, 73, 82, 87, 90, 91, 94, 97, 104, 108, 109, 117, 126, 130, 139, 142, 144, 146–153, 155, 156, 158], aggregating data from 41 independent intervention groups and 2 cross-over groups (34 CR, 2EX and 5 CREX), recruiting 1385 participants. A pooled SMD of –0.17 was found in PYY concentration changes after weight loss (95%CI: –0.28 to –0.06, *p* = 0.002, Fig. 8) with a notable level of heterogeneity among the studies (I^2^ = 68%; *p* < 0.001). Meta-regression analysis did not reveal associations between PYY changes and weight loss magnitude (*p* = 0.081). The asymmetry of the forest plot (SI3 - Fig. S6) and the Egger’s test (z = –3.05, *p* = 0.002) demonstrated potential publication bias.

### Circulating PYY_3-36_ responses in RCTs

2 RCTs [51, 52] with 5 EX intervention groups were included in this meta-analysis, with a total sample size of 97 (SI3 - Fig. S7). A pooled non-significant SMD of 0.40 was found in PYY_3-36_ concentration changes after weight loss (95%CI: –0.22 to 1.03, *p* = 0.206) with a notable level of heterogeneity among the studies (I^2^ = 66%; *p* = 0.025). Meta-regression analysis did not reveal associations between PYY_3-36_ changes and weight loss magnitude (*p* = 0.305). The asymmetry of the forest plot (SI3 - Fig. S8) and the Egger’s test (z = 2.02, *p* = 0.043) demonstrated potential publication bias.

### Circulating PYY_3-36_ responses in non-RCTs

In the analysis of non-RCTs, 8 [73, 92, 93, 98, 115, 128, 143, 149] with 14 intervention groups (8 CR, 5 EX and 1 CREX) were included, recruiting 286 participants (SI3 - Figure S9). A pooled SMD of –0.17 was observed (95%CI: –0.32 to –0.02; *p* = 0.025). The degree of heterogeneity was small and non-significant (I^2^ = 23%, *p* = 0.420). Meta-regression analysis elucidated that each 1% increase in weight loss was associated with a 0.03 decrease in the SMD of PYY_3-36_ concentrations (95%CI: –0.05 to –0.01; *p* = 0.017) and the heterogeneity I^2^ reduced to 0%. The asymmetry in the funnel plot (SI3 - Fig. S10) and the Egger’s test indicate potential publication bias (z = 1.97; *p* = 0.048).

### Circulating total GLP-1 responses in RCTs

4 RCTs [45, 50, 52, 54] were included in the meta-analysis, comprising 8 independent groups across the studies (1 CR; 6 EX; 1 CREX), and involving 233 participants (SI3 - Figure S11). The meta-analysis revealed a pooled non-significant SMD of 0.003 (95% CI: –0.47 to 0.47; *p* = 0.989), along with a moderate degree of between-studies heterogeneity (I^2^ = 57%, *p* = 0.045). According to meta-regression analysis, a 1% increase in weight loss was associated with a 0.08 decrease in the pooled SMD of total GLP-1 concentrations (95% CI: –0.14 to –0.03; *p* = 0.003), which explained 100% of the observed heterogeneity. Potential publication bias was not observed as indicated by the funnel plot and the Egger’s test result (z = –0.20; *p* = 0.844; SI3 - Fig. S12).

### Circulating GLP-1 responses in non-RCTs

This meta-analysis included 22 non-RCTs [73, 82, 87, 90, 98, 99, 111, 121, 142–144, 146–149, 154, 156, 158, 160, 163, 167, 169], aggregating data from 27 independent intervention groups (18 CR, 7 EX; 2 CREX) and comprising 790 individuals (SI3 - Fig. S13). A small and non-significant pooled SMD of –0.07 was reported for the effect of weight loss on total GLP-1 concentration changes (95% CI: –0.20 to 0.06; *p* = 0.264). The analysis revealed a degree of heterogeneity among the included studies (I^2^ = 61%, p < 0.001). Meta-regression analysis did not demonstrate any significant relationships between the magnitude of weight loss and changes in GLP-1 concentrations (p = 0.291). The funnel plot is shown in SI3 - Fig. S14. The Egger’s test was not significant (z = –1.77, *p* = 0.077).

### Circulating active GLP-1 responses in non-RCTs

No RCTs were found exploring active GLP-1 changes after weight loss. 18 non-RCTs [11, 63–65, 69, 70, 73, 74, 76, 86, 91, 93, 96, 103, 109, 153, 161, 164] were included, with 27 independent and 2 cross-over intervention groups (24 CR and 5 EX groups), involving 757 participants. Given a correlation coefficient could not be calculated from the eligible studies, a correlation coefficient of 0.5 was applied and a series of sensitivity analyses were undertaken. A pooled SMD of -0.16 was observed for the effect of weight loss on active GLP-1 concentration changes (95% CI: –0.28 to –0.05; *p* = 0.006; SI3 - Fig. S15) with a moderate degree of heterogeneity among the included studies (I^2^ = 44%, *p* = 0.008). Meta-regression analysis did not find significant associations between weight loss and active GLP-1 changes (*p* = 0.082). The funnel plot and Egger’s test did not indicate significant bias (z = 0.36, *p* = 0.719; SI3 - Figure S16).

### Circulating CCK responses in RCTs and non-RCTs

19 studies [11, 50, 54, 67, 69, 70, 73, 76, 93, 100, 101, 104, 111, 117, 142, 149, 153, 156, 159] were included in this quantitative synthesis, of which 2 studies [50, 54] were RCTs, and 17 studies were non-RCTs. Total sample sizes for RCTs and non-RCTs were 126 and 547, respectively. Of the 2 RCTs, there were 4 independent intervention groups, including 3 EX and 1 CR. The 17 non-RCTs, included 21 independent intervention groups, of which the majority were CR (n = 18), followed by CREX (n = 2) and EX (n = 1). The correlation coefficient was set to 0.5 given the lack of correlation coefficients obtained from the incorporated studies.

Since there were only 2 RCTs [50, 54] with 4 intervention groups, we did not perform a meta-analysis. The study [50] applying EX as a weight loss strategy reported no change of basal CCK while CR [54] resulted in a significant decrease of basal CCK. For non-RCTs, a pooled SMD of -0.18 was observed in changes of CCK concentrations after weight loss (95% CI: -0.37 to 0.02; *p* = 0.076; SI3 - Fig. S17), with a moderate heterogeneity amongst the studies (I^2^ = 74%, *p* < 0.001). No association was identified between the extent of weight loss and changes in CCK concentrations (slope: -0.03, 95% CI: -0.06 to 0.01; *p* = 0.107). The symmetry in the funnel plot and the results from the Egger’s test (z = 0.97, *p* = 0.332; SI3 - Fig. S18) showed no potential publication bias.

### Subgroup analysis: effect of interventions on appetite-hormone changes

Where possible we employed subgroup analysis to gain greater insight into potential differences in the effects of the three interventions (CR, EX, CREX) on appetite related gut hormone concentrations. This was not possible for some outcomes due to an insufficient number of intervention groups (total PYY RCTs, PYY_3-36_ RCTs, total GLP-1 RCTs). These analyses did not reveal significant differences among interventions for each hormone. Additional analyses were conducted to assess the effects while controlling for the extent of weight loss, but the results remained unchanged. Therefore, the results detailed in each individual forest plot are presented without controlling for the extent of weight loss.

### Exploratory analysis for FFM loss, sex, study duration and study design

For body composition changes, the extent of FFM losses correlated positively with increases in total ghrelin for RCTs (slope: 0.21, 95%CI: 0.08–0.34, *p* = 0.002) and non-RCTs (slope: 0.07, 95%CI: 0.004 to 0.14, *p* = 0.038). Conversely, there was a negative correlation between changes in total GLP-1 concentrations and changes in FFM from RCTs (slope: −0.12, 95%CI: –0.20 to–0.04, *p* = 0.002). Similar results were observed when analysing the FFM/FM ratio; however, due to the limited number of studies available for this analysis, these results are not included in the presentation. No differences were identified between studies recruiting females only and males only across all appetite-related hormones (*p* ≥ 0.176). Mixed-sex cohorts exhibited a 0.33 higher increase in total ghrelin from non-RCTs than female only cohorts (*p* = 0.005) when weight loss magnitude was held consistent. A positive association was found between study duration and changes in GLP-1 concentrations in non-RCTs, while controlling for the magnitude of weight loss. Specifically, each additional week of intervention was associated with an increase of 0.05 in the SMD of total GLP-1 (95% CI: 0.0002 to 0.010, *p* = 0.042). No significant differences were identified between RCTs and non-RCTs either overall or in each intervention (*p* ≥ 0.589), except RCTs elicited 0.78 and 0.73 lower SMD for AG in EX and overall interventions, respectively, compared with non-RCTs (*p* = 0.020). For non-RCTs, sensitivity analyses using E-values offers a detailed assessment of the robustness of outcomes in relation to potential unmeasured confounding. Total ghrelin, PYY, total GLP-1 and active GLP-1 demonstrated reasonable robustness to moderate confounding. In contrast, acylated ghrelin, PYY_3-36,_ and CCK displayed some sensitivity to confounding, indicating that unmeasured factors may potentially influence their observed effects.

### Risk of bias assessment

126 studies were included for the risk of bias assessment because one non-RCT study included in the meta-analysis was only reported in abstract form [162] and hence did not contain sufficient details to conduct a risk of bias assessment. The results are provided in SI3 - Figures S19 and S20. Overall, for the RCTs, the risk of bias was low in 12 studies (63%), moderate in 6 studies (32%) and high in one study (5%). For non-RCTs, the risk of bias was low in 36 studies (34%), moderate in 68 studies (63%) with 1 serious (1%) and 2 critical (2%) risks of bias across studies. Sensitivity analyses were conducted after removing the RCT with high-risk of bias and 3 non-RCTs with serious and critical risk of bias. No significant changes were observed in the SMD after removal of the high-risk studies. However, subgroup analysis showed that for total ghrelin, CR exhibited a 0.53 higher SMD increase compared with EX after removing the high-risk of bias RCT [55]. This difference was absent when controlling for the extent of weight loss. The results excluding the studies classified as high risk of bias are not shown in this meta-analysis.

## Discussion

This review summarises: (1) appetite-related gut hormone changes after weight loss in people with overweight or obesity and (2) the differences among interventions involving CR, EX or their combination. This review also identifies potential factors (i.e., loss of FFM, sex, and study duration) that may modify the effect of weight loss on appetite-related gut hormone concentrations. The appetite hormone responses varied by peptide, with an increase in total ghrelin and no changes in total GLP-1 observed in both RCTs and non-RCTs, and inconsistent responses for acylated ghrelin, total PYY and PYY_3-36_. There was limited data for active GLP-1 and CCK in RCTs, with non-RCTs showing a decrease in active GLP-1 and no change in CCK. Greater weight loss is associated with larger increases in total ghrelin concentrations and varied changes in other appetite-related hormones. No significant differences were identified among the interventions i.e., CR, EX and CREX. Exploratory analysis showed that greater FFM loss was correlated with a greater increase in total ghrelin in both RCTs and non-RCTs, and a greater decrease in total GLP-1 in RCTs. Additionally, shorter study duration, given constant weight loss, was correlated with a greater decrease in total GLP-1 in RCTs.

For the orexigenic hormone ghrelin, a statistically significant increase in total ghrelin after weight loss was identified in RCT and non-RCT studies. Furthermore, a greater magnitude of weight loss was associated with a greater increase in total ghrelin concentrations, suggesting a physiological adaptation after weight loss. However, the results for acylated ghrelin were inconsistent. A decrease of acylated ghrelin was observed in RCTs along with a notable correlation indicating that a greater degree of weight loss corresponded with a larger decrease in acylated ghrelin levels. In contrast, the results summarised from non-RCTs indicated that acylated ghrelin increased after weight loss. Experimental research shows that weight loss induced by caloric restriction or exercise primarily increases total ghrelin by raising plasma levels of des-acyl ghrelin, rather than acylated ghrelin, in individuals with overweight or obesity [44]. Our meta-analysis identified an increase in total ghrelin after weight loss but inconsistent results regarding acylated ghrelin concentrations. One potential explanation for the varied findings on acylated ghrelin is the reduced activity of the GOAT enzyme, which is crucial for the acylation process of ghrelin, under conditions of prolonged energy deficiency [170]. The increase in ghrelin induced by weight loss, combined with a possible GOAT-mediated reduction in acylation, may contribute to the variability in acylated ghrelin concentrations, as one effect could outweigh the other. Additionally, the RCTs primarily involved EX interventions, which may further reduce GOAT activity by redistributing blood flow from the splanchnic region to active muscles [171], although long-term effect remains unclear. Findings from non-RCTs also showed sensitivity to unmeasured confounders. However, measuring appetite-related hormones after an overnight fasting helped mitigate some of these confounding effects. Future studies could explore whether calorie restriction in RCTs also has the potential to decrease acylated ghrelin levels. Despite the differing impacts on acylated ghrelin, des-acyl ghrelin has been reported to stimulate food intake independently of the growth hormone secretagogue receptor-1a (GHSR-1a) in rats [172]. Thus, it is possible that an increase in total ghrelin may increase appetite in humans in the absence of an increase in acylated ghrelin. However, it remains to be determined whether an increase in total ghrelin represents a compensatory response to weight loss, reflecting the body’s effort to counterbalance the energy deficit, or if it indicates a return to pre-overweight values, according to the normalisation theory [73].

Analysis of RCTs showed no significant changes in total PYY and PYY_3-36_ concentrations after weight loss, while non-RCTs identified a significant decrease in both. This discrepancy may be due to differences in weight loss extent. Most RCTs reported less than 5% weight loss for total PYY, whereas over half of non-RCTs achieved more than 5%. Similarly, for PYY_3-36_, non-RCTs reported greater weight loss (over 10% in one study with two intervention groups and over 15% in another study), compared to less than 10% in RCTs. Notably, the RCT [47] with resistance training reported the greatest weight loss and the greatest increase in total PYY, contributing to the observed association between greater weight loss and a larger increase in total PYY. Previous research suggests a role for IL-6 in this context, with IL-6 infusion increasing PYY expression [173]. CR or CREX interventions, as opposed to EX alone, have been shown to decrease IL-6 concentrations in people with overweight or obesity, independent of body weight changes [174, 175]. This highlights the potential of exercise in preventing decreases in total PYY during weight loss.

No significant changes in total GLP-1 were observed after weight loss, yet a decrease in active GLP-1 was noted in non-RCTs. Greater weight loss was associated with a greater decrease in total GLP-1 in RCTs, suggesting that changes in this peptide may only become apparent with more substantial weight loss. In addition, the active form of GLP-1 has been recognised for its biological activity. However, recent research has identified that the inactive form, GLP-1_9–37_, may also influence physiological processes, for instance, it may elicit a modest anti-hyperglycaemic effect independent of insulin in humans [176]. This finding suggests that the inactive form of GLP-1 may also have biologically important activity. Therefore, it is critical to determine which form – total or active – responds to a reduced-obesity state.

Regarding CCK, there were not enough RCTs to perform a meta-analysis. The included studies that applied EX indicated no changes in CCK, while CR-induced weight loss led to a decrease in CCK. Both study modes (CR and EX) elicited less than 5% weight loss, suggesting that greater weight loss may be required to elicit changes in CCK. Findings from non-RCTs showed no significant changes in CCK after weight loss. However, sensitivity analyses indicated non-robust results for CCK, suggesting a trend of decreasing CCK concentrations as the correlation coefficient increases and sensitivity to unmeasured confounders.

This is the first meta-analysis that makes direct comparisons between the effects of CR, EX and CREX on appetite related gut hormones. Previous systematic reviews, with or without meta-analysis, have explored the effects of CR and EX separately on appetite-related hormones, suggesting a hormonal response to CR favouring appetite stimulation after weight loss [23, 24], but not EX [25]. Our results showed no differences among interventions for any appetite-related hormone, except that CR led to a greater increase in total ghrelin compared with EX after removing the high-risk of bias RCT study. However, when controlling for the magnitude of weight loss, the variations in total ghrelin changes across different interventions were rendered non-significant. This finding suggests that the differences among interventions might predominantly stem from differing degrees of weight loss. In addition, the results for each intervention, as shown in the forest plot, indicate that CR was associated with a significant increase in total ghrelin and significant decreases in total PYY, PYY_3-36_, and active GLP-1 after weight loss, while EX elicited a significant decrease in acylated ghrelin and no significant changes in any other hormone. These findings are consistent with the aforementioned reviews [23, 24] that assessed CR and EX separately. However, when examining the extent of weight loss, more CR studies achieved 10–15% or 15–20% weight loss, whereas the EX studies tended to elicit less than 5% or between 5 and 10% weight loss. Therefore, differences in weight loss magnitude could be the reason driving the differences observed in previous reviews and our results for each intervention. The impact of CREX on appetite-related hormones remains uncertain due to the limited number of studies.

In addition, CR and EX might lead to different results due to varied changes in body composition [177]. FFM is considered to influence hormone concentrations. It has been demonstrated that weight loss resulting in FFM deficits leads to higher total [178] and acylated ghrelin [179] concentrations. Our results show that greater FFM loss is correlated with a greater total ghrelin increase in both RCTs and non-RCTs and greater reductions in total GLP-1 concentrations in RCTs. Given the nature of the interventions in the included studies, which predominantly involve CR and aerobic training, these approaches are typically associated with a greater proportion of weight loss coming from FFM [180]. Aerobic training alone is typically less effective in preserving FFM compared with resistance training [177]. Studies in this meta-analysis revealed a significant association between FFM loss and overall weight loss, making it challenging to discern whether observed differences were due to weight loss or FFM loss. This highlights the need for future research that emphasises FFM preservation while promoting fat loss to better elucidate the relationship between FFM and appetite-related hormones.

We also investigated the impact of sex and the rate of weight loss (determined by accounting for study duration) on appetite-related hormone changes while keeping the magnitude of weight loss constant. Research has indicated sex-specific effects on certain appetite-related hormones. For example, active GLP-1 concentrations have been reported to decrease in men, but not in women, after weight loss induced by a very-low-calorie ketogenic diet [153]. Our analysis from non-RCTs revealed that mixed-sex cohorts had greater increases in total ghrelin after weight loss than female-only cohorts, when the extent of weight loss was controlled for.

This finding suggests a potentially stronger effect of weight loss on increasing total ghrelin levels in men than in women. A possible explanation is that men typically have higher baseline FFM and are therefore likely to experience more substantial FFM reductions during weight loss, as predicted by the Forbes curve [181, 182]. This hypothesis is supported by our data: studies with mixed cohorts reported a mean baseline body fat percentage of 41%, compared to 45% in female-only studies, with an average FFM loss of 1.7 kg in mixed cohorts versus 1.1 kg in female-only groups. Additionally, the proportion of FM/FFM loss was 3.5 in mixed cohorts and 3.9 in female-only groups, suggesting a higher FFM loss in mixed cohort than in female groups. The absence of significant differences between male and female-only groups in our analysis might stem from the limited number of studies that recruited exclusively male or female participants, potentially leading to underpowered results. The positive correlation between study duration and total GLP-1 changes when controlling for total weight loss suggests that a slower rate of weight loss may be beneficial in preventing decreases in total GLP-1 levels possibly due to preservation of FFM in current study when the rate of weight loss is slower. Previous research indicates no significant differences in fasting acylated ghrelin, total PYY, active GLP-1, and CCK between rapid and gradual weight loss when the amount of weight loss is controlled for [70]. This is possibly due to changes in body composition being similar regardless of the rate of weight loss provided that total weight loss is similar [183]. Further research is warranted to investigate whether these factors influence appetite-related hormone responses and how this might relate to long-term weight maintenance.

One strength of this meta-analysis is its inclusion of a large number of studies, enabling a comprehensive analysis and the identification of potential modifiers, such as the magnitude of weight loss and body composition changes. However, this breadth also introduces substantial heterogeneity, even though some of the observed heterogeneity was explained by the identified modifiers. The inclusion of studies with varied protocols may raise questions about the rationale behind combining these studies. Unfortunately, while some studies employed specific protocols (e.g., MICT, resistance training, and CR variants like ketogenic diets), we had fewer than five independent groups for many specific protocols. Where feasible, we conducted protocol-specific meta-analyses, and most findings were generally consistent with those from the combined analyses. For many hormones, high heterogeneity persisted even within protocol-specific analyses with larger sample sizes, suggesting that factors beyond intervention protocols—such as measurement variability and natural hormonal fluctuations among participants—may contribute to the heterogeneity observed. We also noted some protocol-specific effects; for instance, resistance training was associated with decreases in AG, while high-fibre diets were linked to increases in CCK. Future studies could investigate the underlying mechanisms driving these protocol-specific responses to provide a clearer understanding of the factors influencing appetite-related hormone changes.

In conclusion, this meta-analysis demonstrates that weight loss triggers a consistent increase in total ghrelin and no changes in total GLP-1, while the results for other appetite-related hormones are variable. The inconsistent results may be related to the amount of weight loss. Additionally, no differences were found among interventions when the amount of weight loss was controlled for. These findings emphasize the necessity for more RCTs of CR and CREX to directly compare various weight-loss interventions and explain potential modifying factors, such as loss of FFM. A key focus for future research should be to determine whether these hormonal responses are predictors of compensation, possibly through mediation analyses examining changes in daily energy intake, or if they represent a normalization to pre-overweight levels.

## Supplementary information

Search terms

Correlation coefficients

Supplementary information content 3

Supplementary Table 1

## Data Availability

The data presented in this study are accessible through the Loughborough University Research Repository. https://repository.lboro.ac.uk/articles/dataset/Fasting_appetite-related_gut_hormone_responses_after_weight_loss_induced_by_calorie_restriction_exercise_or_both_in_people_with_overweight_or_obesity_a_meta_analysis_/28142897/2?file=51499148.
