# Supplementary material for: Fasting appetite-related gut hormone responses after weight loss induced by calorie restriction, exercise, or both in people with overweight or obesity: a meta‐analysis
Source: Int J Obes (Lond). 2025 Feb 10;49(5):776–92. doi: 10.1038/s41366-025-01726-4 (PMC12095072; doi:10.1038/s41366-025-01726-4)
Supplement: Supplementary file 1 — Search terms [file 41366_2025_1726_MOESM1_ESM.pdf]

Cochrane

#1	(“weight loss diet” OR “weight reduction diet” OR “low calori*” OR “calorie restrict*” OR “caloric restrict*” OR “hypocaloric” OR “low energy” OR “energy deficit” OR “energy restrict*” OR “low carbohydrate” OR “carbohydrate restrict*” OR “low fat” OR “fat restrict*” OR ketogenic OR “high protein” OR “mediterranean diet” OR “vegetarian diet” OR “plant-based diet”):ti,ab,kw	
#2	MeSH descriptor: [Diet] explode all trees	
#3	MeSH descriptor: [Diet, Reducing] explode all trees	
#4	MeSH descriptor: [Caloric Restriction] explode all trees	
#5	MeSH descriptor: [Diet, Carbohydrate-Restricted] explode all trees	
#6	MeSH descriptor: [Diet, Fat-Restricted] explode all trees	
#7	MeSH descriptor: [Diet, High-Protein] explode all trees	
#8	MeSH descriptor: [Feeding Behavior] this term only	
#9	(“physical activity” OR exercise OR “resistance training” OR “resistance exercise” OR “aerobic training” OR “aerobic exercise” OR “strength training” OR “plyometric exercise” OR “endurance training” OR “endurance exercise” OR “high-intensity exercise” OR “high-intensity exercise” OR “moderate-intensity training” OR “moderate-intensity exercise” OR “low-intensity training” OR “low-intensity exercise” OR sport OR run* OR cycl* OR walk* OR swim*):ti,ab,kw	
#10	MeSH descriptor: [Exercise] explode all trees	
#11	MeSH descriptor: [Resistance Training] explode all trees	
#12	MeSH descriptor: [Plyometric Exercise] explode all trees	
#13	MeSH descriptor: [Endurance Training] explode all trees	
#14	MeSH descriptor: [High-Intensity Interval Training] explode all trees	
#15	(“gastrointestinal hormone” OR “gut peptide” OR “gut hormone” OR “peptide hormone” OR “appetite hormone” OR “appetite related hormone” OR “appetite regulating hormone”):ti,ab,kw	
#16	(“gastrointestinal hormones” OR “gut peptides” OR “gut hormones” OR “peptide hormones” OR “appetite hormones” OR “appetite related hormones” OR “appetite regulating hormones”):ti,ab,kw	
#17	(ghrelin OR “acylated ghrelin” OR “acyl ghrelin” OR “total ghrelin” OR “desacylated ghrelin” OR “desacyl ghrelin” OR “peptide YY” OR PYY OR “glucagon-like peptide 1” OR GLP-1 OR cholecystokinin OR CCK OR “pancreatic polypeptide” OR oxyntomodulin OR OXM):ti,ab,kw	
filter	trials	
	(#1 OR #2 OR #3 OR #4 OR #5 OR #6 OR #7 OR #8) AND (#15 OR #16 OR #17)	967
	(#9 OR #10 OR #11 OR #12 OR #13 OR #14) AND (#15 OR #16 OR #17)	994

Embase

#1	weight loss diet':ti,ab,kw OR 'weight reduction diet':ti,ab,kw OR 'low calori*':ti,ab,kw OR 'calorie restrict*':ti,ab,kw OR 'caloric restrict*':ti,ab,kw OR 'hypocaloric':ti,ab,kw OR 'low energy':ti,ab,kw OR 'energy deficit':ti,ab,kw OR 'energy restrict*':ti,ab,kw OR 'low carbohydrate':ti,ab,kw OR 'carbohydrate restrict*':ti,ab,kw OR 'low fat':ti,ab,kw OR 'fat restrict*':ti,ab,kw OR 'ketogenic':ti,ab,kw OR 'high protein':ti,ab,kw OR 'mediterranean diet':ti,ab,kw OR 'vegetarian diet':ti,ab,kw OR 'plant-based diet':ti,ab,kw OR 'diet'/exp OR 'caloric restriction'/exp OR 'low calorie diet'/exp OR 'low carbohydrate diet'/exp OR 'low fat diet'/exp OR 'ketogenic diet'/exp OR 'protein diet'/exp OR 'feeding behavior'/exp	
#2	physical activity':ti,ab,kw OR exercise:ti,ab,kw OR 'resistance training':ti,ab,kw OR 'resistance exercise':ti,ab,kw OR 'aerobic training':ti,ab,kw OR 'aerobic exercise':ti,ab,kw OR 'strength training':ti,ab,kw OR 'plyometric exercise':ti,ab,kw OR 'endurance training':ti,ab,kw OR 'endurance exercise':ti,ab,kw OR 'high-intensity training':ti,ab,kw OR 'high-intensity exercise':ti,ab,kw OR 'moderate-intensity training':ti,ab,kw OR 'moderate-intensity exercise':ti,ab,kw OR 'low-intensity training':ti,ab,kw OR 'low-intensity exercise':ti,ab,kw OR sport:ti,ab,kw OR run*:ti,ab,kw OR cycl*:ti,ab,kw OR walk*:ti,ab,kw OR swim*:ti,ab,kw OR 'exercise'/exp OR 'resistance training'/exp OR 'plyometrics'/exp OR 'endurance training'/exp OR 'high intensity interval training'/exp	
#3	gastrointestinal hormone*':ti,ab,kw OR 'gut peptide*':ti,ab,kw OR 'gut hormone*':ti,ab,kw OR 'peptide hormone*':ti,ab,kw OR 'appetite hormone*':ti,ab,kw OR 'appetite related hormone*':ti,ab,kw OR 'appetite regulating hormone*':ti,ab,kw OR ghrelin:ti,ab,kw OR 'acylated ghrelin':ti,ab,kw OR 'acyl ghrelin':ti,ab,kw OR 'total ghrelin':ti,ab,kw OR 'desacylated ghrelin':ti,ab,kw OR 'desacyl ghrelin':ti,ab,kw OR 'peptide YY':ti,ab,kw OR PYY:ti,ab,kw OR 'glucagon-like peptide 1':ti,ab,kw OR 'GLP-1':ti,ab,kw OR cholecystokinin:ti,ab,kw OR CCK:ti,ab,kw OR 'pancreatic polypeptide':ti,ab,kw OR oxyntomodulin:ti,ab,kw OR OXM:ti,ab,kw	
filter	([adult]/lim OR [aged]/lim OR [middle aged]/lim OR [very elderly]/lim OR [young adult]/lim)	
	('clinical trial'/de OR 'controlled study'/de OR 'randomized controlled trial'/de OR 'controlled clinical trial')	
	human'/de	
	#1 AND #3	1625
	#2 AND #3	1515

Pubmed

#1	("diet"[MeSH Terms] OR "weight loss diet"[Title/Abstract] OR "weight reduction diet"[Title/Abstract] OR "diet, reducing"[MeSH Terms] OR "low calori*"[Title/Abstract] OR "calorie restrict*"[Title/Abstract] OR "caloric restrict*"[Title/Abstract] OR "hypocaloric"[Title/Abstract] OR "low energy"[Title/Abstract] OR "energy deficit"[Title/Abstract] OR "energy restrict*"[Title/Abstract] OR "caloric restriction"[MeSH Terms] OR "low carbohydrate"[Title/Abstract] OR "carbohydrate restrict*"[Title/Abstract] OR "diet, carbohydrate restricted"[MeSH Terms] OR "low fat"[Title/Abstract] OR "fat restrict*"[Title/Abstract] OR "diet, fat restricted"[MeSH Terms] OR ketogenic[Title/Abstract] OR "high protein"[Title/Abstract] OR "mediterranean diet"[Title/Abstract] OR "vegetarian diet"[Title/Abstract] OR "diet, high protein low carbohydrate"[MeSH Terms] OR "diet, high protein"[MeSH Terms] OR "diet, ketogenic"[MeSH Terms] OR "diet, mediterranean"[MeSH Terms] OR "plant-based diet"[Title/Abstract] OR "diet, vegetarian"[MeSH Terms] OR "feeding behavior"[MeSH Terms])	
#2	("physical activity"[Title/Abstract] OR "exercise"[Title/Abstract] OR "Exercise"[MeSH Terms] OR "resistance exercise"[Title/Abstract] OR "resistance training"[Title/Abstract] OR "Resistance Training"[MeSH Terms] OR "aerobic training"[Title/Abstract] OR "aerobic exercise"[Title/Abstract] OR "strength training"[Title/Abstract] OR "plyometric exercise"[Title/Abstract] OR "Plyometric Exercise"[MeSH Terms] OR "endurance exercise"[Title/Abstract] OR "endurance training"[Title/Abstract] OR "Endurance Training"[MeSH Terms] OR "high-intensity training"[Title/Abstract] OR "high-intensity exercise"[Title/Abstract] OR "High-Intensity Interval Training"[MeSH Terms] OR "moderate-intensity training"[Title/Abstract] OR "moderate-intensity exercise"[Title/Abstract] OR "low-intensity training"[Title/Abstract] OR "low-intensity exercise"[Title/Abstract] OR "sport"[Title/Abstract] OR "run"[Title/Abstract] OR "running"[Title/Abstract] OR "cycle"[Title/Abstract] OR "cycling"[Title/Abstract] OR "walk"[Title/Abstract] OR "walking"[Title/Abstract] OR "swim"[Title/Abstract] OR "swimming"[Title/Abstract])	
#3	("gastrointestinal hormone*"[Title/Abstract] OR "gut peptide*"[Title/Abstract] OR "gut hormone*"[Title/Abstract] OR "peptide hormone*"[Title/Abstract] OR "appetite hormone*"[Title/Abstract] OR "appetite related hormone*"[Title/Abstract] OR "appetite regulating hormone*"[Title/Abstract] OR "ghrelin"[Title/Abstract] OR "acylated ghrelin"[Title/Abstract] OR "acyl ghrelin"[Title/Abstract] OR "total ghrelin"[Title/Abstract] OR "desacylated ghrelin"[Title/Abstract] OR "desacyl ghrelin"[Title/Abstract] OR "peptide YY"[Title/Abstract] OR "PYY"[Title/Abstract] OR "glucagon-like peptide 1"[Title/Abstract] OR "GLP-1"[Title/Abstract] OR "cholecystokinin"[Title/Abstract] OR "CCK"[Title/Abstract] OR "pancreatic polypeptide"[Title/Abstract] OR "oxyntomodulin"[Title/Abstract] OR "OXM"[Title/Abstract])	
Filter	Clinical Trial, Randomized Controlled Trial	
	#1 AND #3	972
	#2 AND #3	342

SPORTDiscus

1	diet* OR weight loss diet OR weight reduction diet OR reducing diet OR low calori* OR calorie restrict* OR caloric restrict* OR hypocaloric OR low energy OR energy deficit OR energy restrict* OR low carbohydrate OR carbohydrate restrict* OR low fat OR fat restrict* OR ketogenetic OR high protein OR mediterranean diet OR vegetarian diet OR plant-based diet OR feeding behavio?r		
2	physical activity OR exercise OR resistance training OR resistance exercise OR aerobic training OR aerobic exercise OR strength training OR plyometric exercise OR endurance training OR endurance exercise OR high-intensity training OR high-intensity exercise OR moderate-intensity training OR moderate-intensity exercise OR low-intensity training OR low-intensity exercise OR sport OR run* OR cycl* OR walk* OR swim*		
3	gastrointestinal hormone* OR gut peptide* OR gut hormone* OR peptide hormone* OR appetite hormone* OR appetite related hormone* OR appetite regulating hormone* OR ghrelin OR acylated ghrelin OR acyl ghrelin OR total ghrelin OR desacylated ghrelin OR desacyl ghrelin OR peptide YY OR PYY OR glucagon-like peptide 1 OR GLP-1 OR cholecystokinin OR CCK OR pancreatic polypeptide OR oxyntomodulin OR OXM		
	filter: academic journal	abstract	title
	1 AND 3	473	52
	2 AND 3	360	91
	N.B. To export, click share, results (1-50), select next page, results (51-100) etc until all pages in results folder, click export on the right hand side and export as RIS format.		

Web of Science

1	(diet* OR “weight loss diet” OR “weight reduction diet” OR “reducing diet” OR “low calori*” OR “calorie restrict*” OR “caloric restrict*” OR “hypocaloric” OR “low energy” OR “energy deficit” OR “energy restrict*” OR “low carbohydrate” OR “carbohydrate restrict*” OR “low fat” OR “fat restrict*” OR ketogenetic OR “high protein” OR “mediterranean diet” OR “vegetarian diet” OR “plant-based diet” OR “feeding behavio?r”)	Topic
2	(“physical activity” OR exercise OR “resistance training” OR “resistance exercise” OR “aerobic training” OR “aerobic exercise” OR “strength training” OR “plyometric exercise” OR “endurance training” OR “endurance exercise” OR “high-intensity training” OR “high-intensity exercise” OR “moderate-intensity training” OR “moderate-intensity exercise” OR “low-intensity training” OR “low-intensity exercise” OR sport OR run* OR cycl* OR walk* OR swim*)	Topic
3	(“gastrointestinal hormone*” OR “gut peptide*” OR “gut hormone*” OR “peptide hormone*” OR “appetite hormone*” OR “appetite related hormone*” OR “appetite regulating hormone*” OR ghrelin OR “acylated ghrelin” OR “acyl ghrelin” OR “total ghrelin” OR “desacylated ghrelin” OR “desacyl ghrelin” OR “peptide YY” OR PYY OR “glucagon-like peptide 1” OR GLP-1 OR cholecystokinin OR CCK OR “pancreatic polypeptide” OR oxyntomodulin OR OXM)	Topic
4	(“clinical trial*” OR “controlled trial*” OR “follow-up stud*” OR “prospective stud*” OR random* OR placebo* OR “single blind*” OR “double blind*”)	All fields
	1 AND 3 AND 4	1951
	2 AND 3 AND 4	1207
	N.B. exclude reviews after running the search	

Clinial trials

#1	diet* OR “weight loss diet” OR “calori* restriction” OR hypocaloric OR “low calorie” OR “low carbohydrate” OR “carbohydrate restrict*” OR “low fat” OR “fat restrict*” OR ketogenetic OR “high protein” OR “mediterranean diet” OR “plant-based diet” OR “feeding behavio?r”	Intervention/treatment
#2	“physical activity” OR exercise OR training OR “resistance exercise” OR “aerobic exercise” OR “strength exercise” OR “plyometric exercise” OR “endurance exercise” OR “high-intensity training” OR “moderate-intensity training” OR “low-intensity training” OR sport OR run* OR cycl* OR walk* OR swim*	Intervention/treatment
#3	“gastrointestinal hormone*” OR “gut peptide*” OR “gut hormone*” OR “peptide hormone*” OR “appetite hormone*” OR ghrelin OR “peptide YY” OR “glucagon-like peptide 1” OR cholecystokinin OR “pancreatic polypeptide” OR oxyntomodulin	Outcome measure
filter	completed study/adults/older adult (65+)	
	#1 AND #3	150
	#2 AND #3	157
