# Supplementary material for: Fasting appetite-related gut hormone responses after weight loss induced by calorie restriction, exercise, or both in people with overweight or obesity: a meta‐analysis
Source: Int J Obes (Lond). 2025 Feb 10;49(5):776–92. doi: 10.1038/s41366-025-01726-4 (PMC12095072; doi:10.1038/s41366-025-01726-4)
Supplement: Supplementary file 2 — Correlation coefficients [file 41366_2025_1726_MOESM2_ESM.pdf]

	Correlation coefficient	SMD	ci.lb	ci.ub	p value	Q	I.square
total ghrelin RCTs	0.10	0.35	0.02	0.67	0.04	41.84	76.29
	0.20	0.36	0.03	0.69	0.03	42.99	77.32
	0.30	0.37	0.03	0.72	0.03	44.41	78.51
	0.40	0.39	0.04	0.74	0.03	46.19	79.92
	0.50	0.41	0.04	0.78	0.03	48.51	81.60
	0.60	0.44	0.05	0.83	0.03	51.64	83.65
	0.70	0.49	0.06	0.91	0.02	56.16	86.17
	0.80	0.55	0.07	1.04	0.02	63.36	89.34
	0.90	0.69	0.08	1.30	0.03	77.65	93.38
total ghrelin non-RCTs	0.1	0.19	0.11	0.27	0.00	322.00	72.93
	0.2	0.19	0.11	0.27	0.00	331.37	73.75
	0.3	0.19	0.11	0.28	0.00	342.99	74.71
	0.4	0.20	0.11	0.28	0.00	357.78	75.84
	0.5	0.21	0.12	0.29	0.00	377.31	77.21
	0.6	0.21	0.12	0.31	0.00	404.38	78.89
	0.7	0.23	0.13	0.32	0.00	444.70	81.05
	0.8	0.24	0.14	0.35	0.00	512.33	83.94
	0.9	0.28	0.16	0.41	0.00	656.41	88.20
acylated ghrelin RCTs	0.10	-0.46	-0.86	-0.06	0.02	20.00	55.67
	0.20	-0.49	-0.91	-0.06	0.02	21.94	59.97
	0.30	-0.52	-0.97	-0.06	0.03	24.31	64.32
	0.40	-0.55	-1.04	-0.06	0.03	27.28	68.73
	0.50	-0.60	-1.13	-0.06	0.03	31.14	73.21
	0.60	-0.66	-1.25	-0.06	0.03	36.38	77.76
	0.70	-0.74	-1.42	-0.06	0.03	43.99	82.43
	0.80	-0.88	-1.69	-0.06	0.04	56.46	87.26
	0.90	-1.15	-2.24	-0.05	0.04	82.60	92.39
acylated ghrelin non-RCTs	0.1	0.12	0.02	0.22	0.01	46.64	32.68
	0.2	0.13	0.03	0.23	0.01	51.18	37.19
	0.3	0.13	0.03	0.24	0.01	56.81	42.05
	0.4	0.14	0.03	0.26	0.01	63.98	47.30
	0.5	0.15	0.03	0.28	0.02	73.45	52.98
	0.6	0.17	0.03	0.30	0.02	86.56	59.17
	0.7	0.19	0.04	0.34	0.02	106.01	65.98
	0.8	0.22	0.04	0.39	0.01	138.17	73.61
	0.9	0.29	0.06	0.51	0.01	203.68	82.61
total PYY RCTs	0.1	0.07	-0.56	0.70	0.82	16.91	73.57
	0.2	0.08	-0.59	0.74	0.82	18.69	76.19
	0.3	0.09	-0.62	0.80	0.81	20.89	78.83
	0.4	0.10	-0.67	0.86	0.80	23.67	81.49
	0.5	0.11	-0.72	0.94	0.80	27.32	84.17
	0.6	0.13	-0.80	1.05	0.79	32.31	86.88
	0.7	0.15	-0.90	1.21	0.77	39.57	89.62
	0.8	0.20	-1.06	1.46	0.75	51.18	92.42
	0.9	0.31	-1.36	1.97	0.72	73.42	95.32

total PYY non-RCTs	0.1	-0.12	-0.21	-0.03	0.01	76.89	54.05
	0.2	-0.13	-0.22	-0.04	0.01	79.35	55.58
	0.3	-0.13	-0.23	-0.04	0.01	82.49	57.37
	0.4	-0.14	-0.24	-0.04	0.00	86.62	59.48
	0.5	-0.15	-0.25	-0.05	0.00	92.30	62.02
	0.6	-0.16	-0.27	-0.06	0.00	100.55	65.12
	0.7	-0.18	-0.29	-0.07	0.00	113.64	69.04
	0.8	-0.21	-0.33	-0.08	0.00	137.57	74.24
	0.9	-0.26	-0.41	-0.11	0.00	196.18	81.76
total PYY3-36 RCTs	0.1	0.23	-0.12	0.58	0.20	3.69	29.73
	0.2	0.24	-0.13	0.61	0.20	4.12	33.98
	0.3	0.26	-0.14	0.65	0.20	4.65	38.64
	0.4	0.28	-0.15	0.70	0.20	5.36	43.77
	0.5	0.30	-0.16	0.76	0.20	6.31	49.44
	0.6	0.33	-0.18	0.85	0.20	7.69	55.76
	0.7	0.38	-0.21	0.96	0.20	9.84	62.85
	0.8	0.45	-0.25	1.15	0.21	13.73	70.91
	0.9	0.57	-0.36	1.50	0.23	23.03	80.19
total PYY3-36 non-RCTs	0.1	-0.14	-0.27	-0.01	0.04	8.58	10.42
	0.2	-0.14	-0.27	-0.01	0.04	8.80	10.90
	0.3	-0.14	-0.27	-0.01	0.04	9.09	11.58
	0.4	-0.15	-0.28	-0.01	0.03	9.49	12.59
	0.5	-0.15	-0.29	-0.02	0.03	10.07	14.13
	0.6	-0.16	-0.30	-0.02	0.03	10.94	16.58
	0.7	-0.17	-0.31	-0.02	0.03	12.42	20.78
	0.8	-0.18	-0.34	-0.02	0.03	15.34	28.70
	0.9	-0.21	-0.40	-0.02	0.03	23.40	45.91
total GLP-1 RCTs	0.1	0.02	-0.24	0.29	0.87	3.69	10.48
	0.2	0.02	-0.26	0.30	0.89	4.13	14.37
	0.3	0.02	-0.28	0.31	0.90	4.69	18.84
	0.4	0.02	-0.30	0.33	0.92	5.42	24.02
	0.5	0.01	-0.32	0.35	0.94	6.43	30.12
	0.6	0.01	-0.35	0.37	0.96	7.89	37.43
	0.7	0.01	-0.40	0.41	0.98	10.22	46.36
	0.8	0.00	-0.47	0.48	0.99	14.51	57.57
	0.9	0.00	-0.61	0.62	0.99	25.08	72.13
total GLP-1 non-RCTs	0.1	-0.07	-0.17	0.03	0.17	42.41	36.55
	0.2	-0.07	-0.17	0.03	0.17	43.34	38.07
	0.3	-0.07	-0.17	0.03	0.17	44.52	39.92
	0.4	-0.07	-0.18	0.03	0.17	46.05	42.22
	0.5	-0.08	-0.18	0.03	0.17	48.12	45.14
	0.6	-0.08	-0.19	0.03	0.17	51.09	48.95
	0.7	-0.08	-0.20	0.04	0.17	55.67	54.08
	0.8	-0.09	-0.22	0.04	0.17	63.71	61.31
	0.9	-0.11	-0.26	0.05	0.19	81.75	72.30

active GLP-1 non-RCTs	0.1	-0.13	-0.23	-0.03	0.01	35.34	29.25
	0.2	-0.14	-0.24	-0.04	0.01	37.70	32.38
	0.3	-0.15	-0.25	-0.04	0.01	40.59	35.94
	0.4	-0.15	-0.26	-0.04	0.01	44.23	40.03
	0.5	-0.16	-0.28	-0.05	0.01	48.95	44.71
	0.6	-0.17	-0.30	-0.05	0.01	55.37	50.11
	0.7	-0.19	-0.33	-0.05	0.01	64.73	56.39
	0.8	-0.21	-0.37	-0.06	0.01	80.00	63.79
	0.9	-0.25	-0.44	-0.07	0.01	111.92	72.89
CCK non-RCTs	0.1	-0.13	-0.30	0.03	0.10	50.47	64.11
	0.2	-0.14	-0.31	0.03	0.10	52.80	66.43
	0.3	-0.15	-0.33	0.02	0.09	55.68	68.91
	0.4	-0.16	-0.35	0.02	0.08	59.38	71.56
	0.5	-0.18	-0.37	0.02	0.08	64.28	74.40
	0.6	-0.20	-0.41	0.01	0.07	71.13	77.47
	0.7	-0.22	-0.45	0.00	0.05	81.47	80.83
	0.8	-0.27	-0.53	-0.01	0.04	99.19	84.63
	0.9	-0.36	-0.68	-0.04	0.03	138.45	89.32

SMD: Standardised mean difference; ci.lb: lower bound of the 95% confidence interval; ci.ub: upper bound
