# Supplementary material for: Fasting appetite-related gut hormone responses after weight loss induced by calorie restriction, exercise, or both in people with overweight or obesity: a meta‐analysis
Source: Int J Obes (Lond). 2025 Feb 10;49(5):776–92. doi: 10.1038/s41366-025-01726-4 (PMC12095072; doi:10.1038/s41366-025-01726-4)
Supplement: Supplementary file 3 — Supplementary information content 3 [file 41366_2025_1726_MOESM3_ESM.pdf]

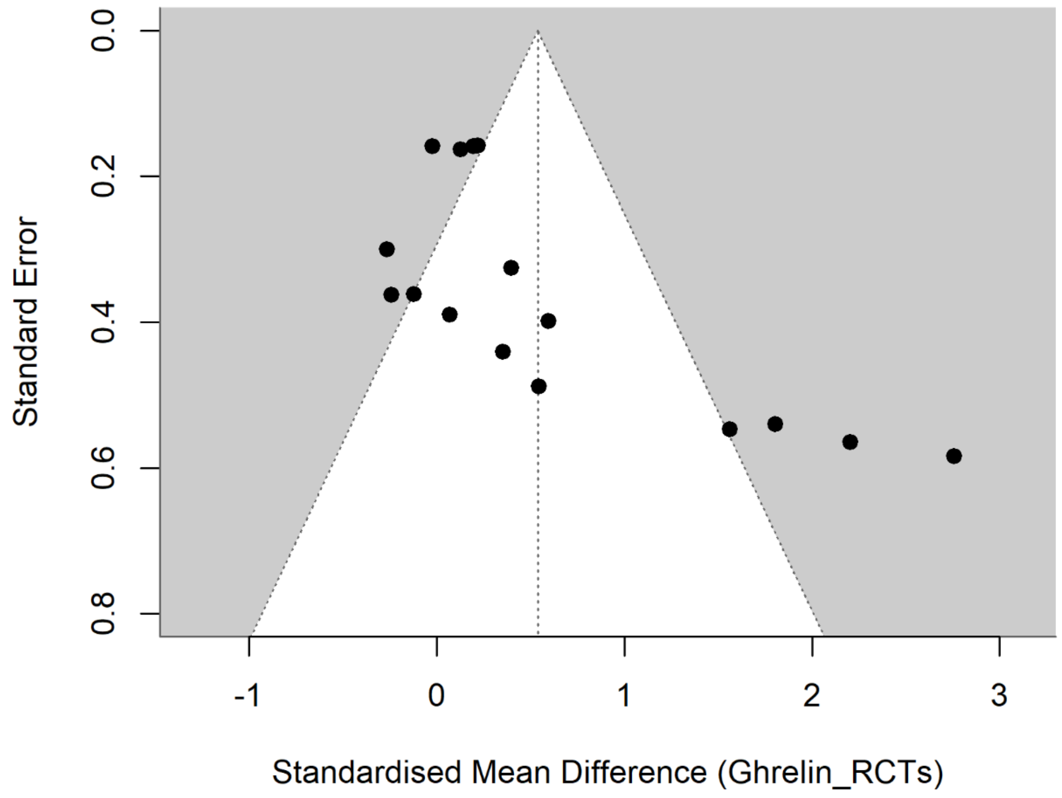

Figure S1. Funnel plot of total ghrelin changes after weight loss from RCTs.

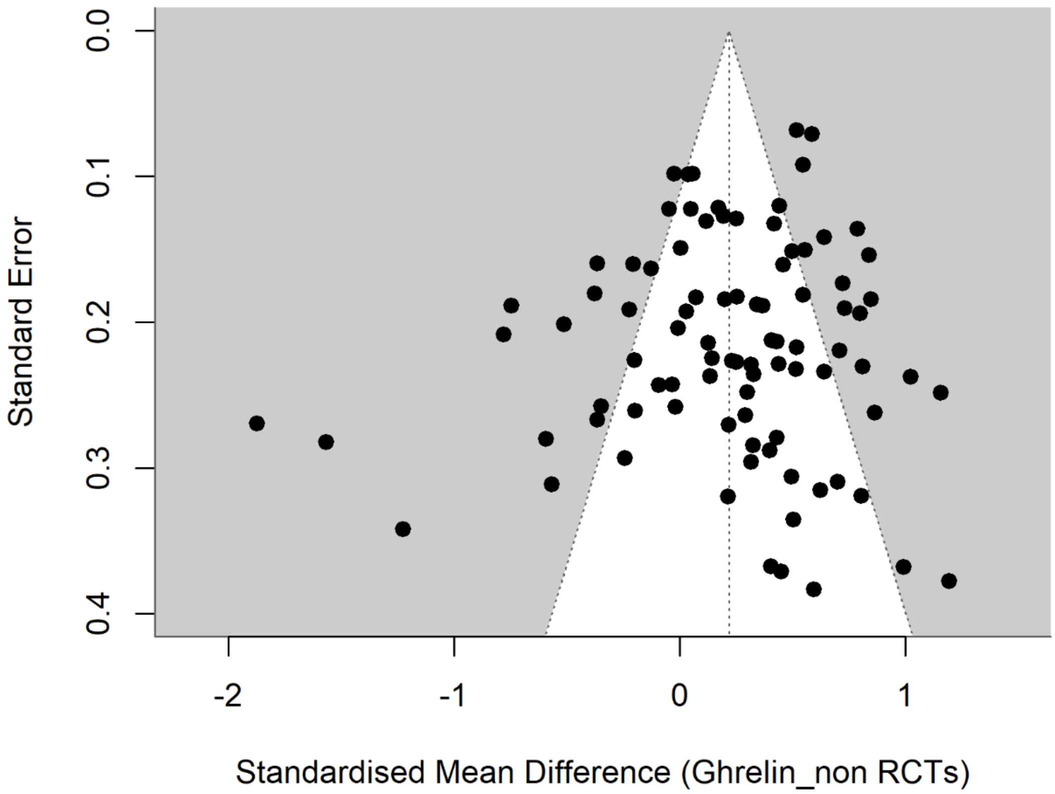

Figure S2. Funnel plot of total ghrelin changes after weight loss from non-RCTs.

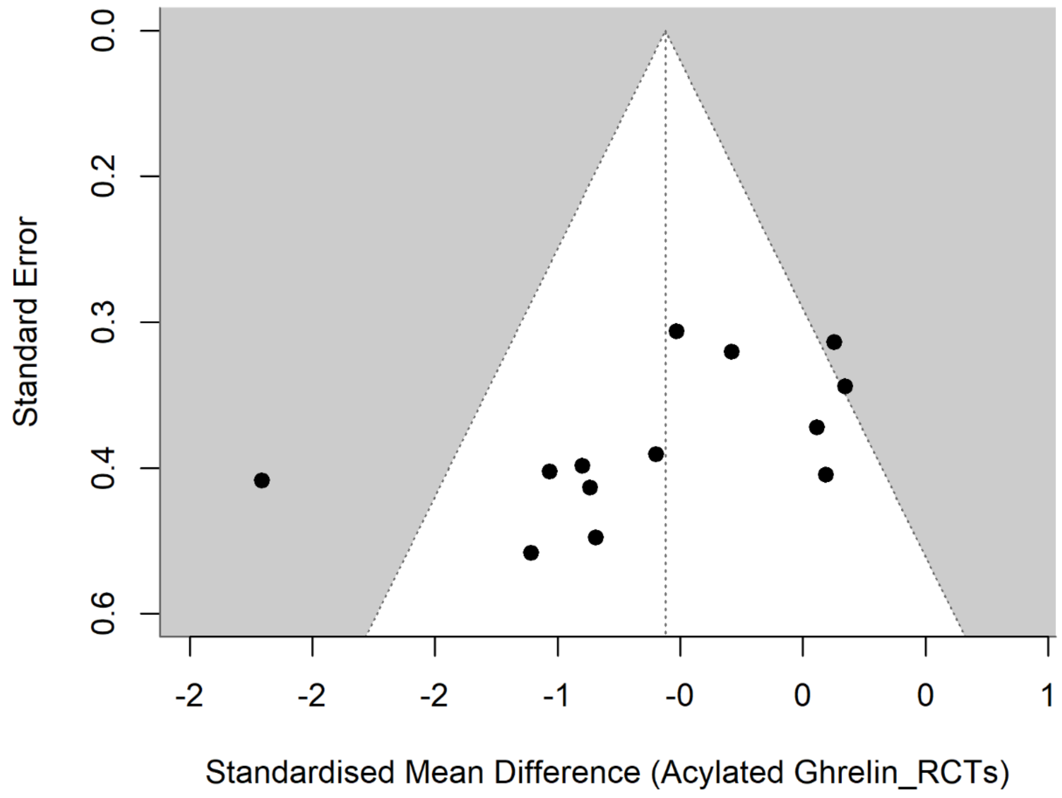

Figure S3. Funnel plot of acylated ghrelin changes after weight loss from RCTs.

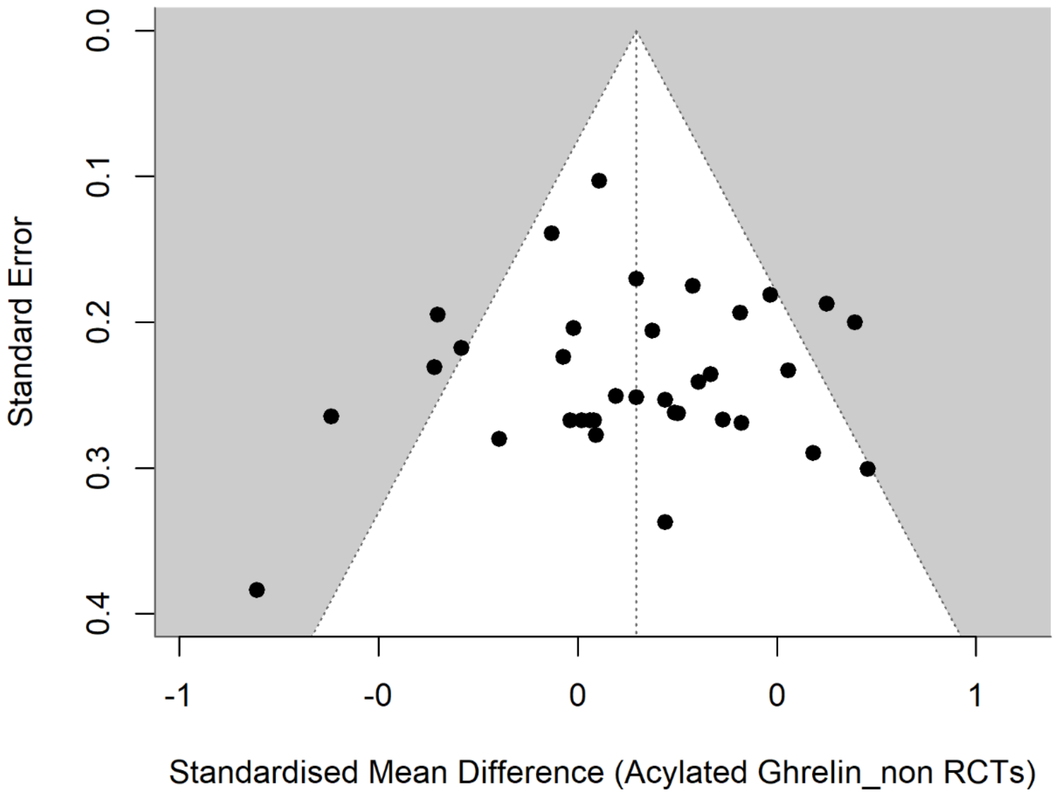

Figure S4. Funnel plot of acylated ghrelin changes after weight loss from non-RCTs.

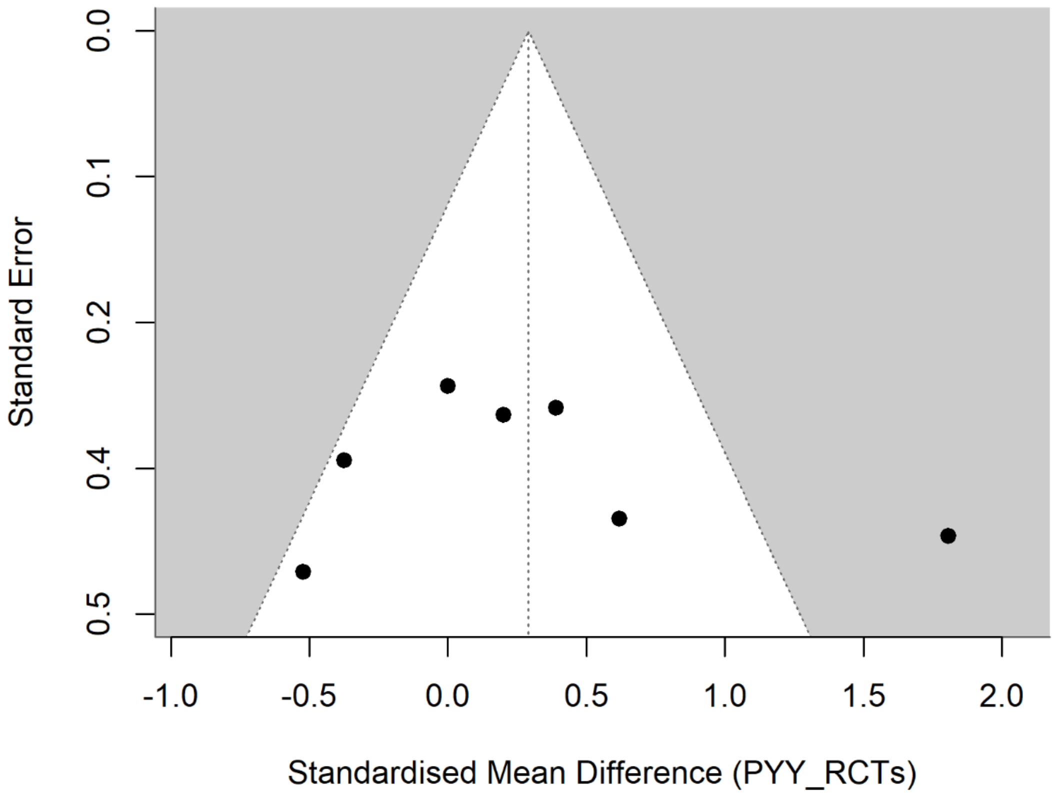

Figure S5. Funnel plot of total PYY changes after weight loss from RCTs.

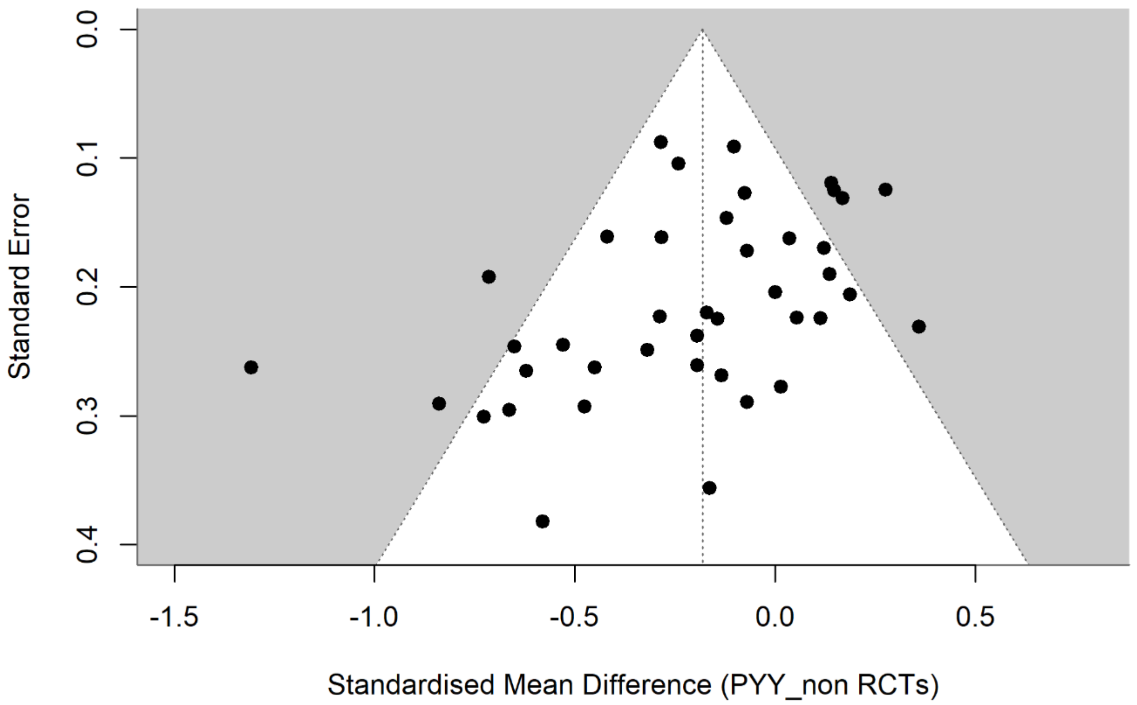

Figure S6. Funnel plot of total PYY changes after weight loss from non-RCTs.

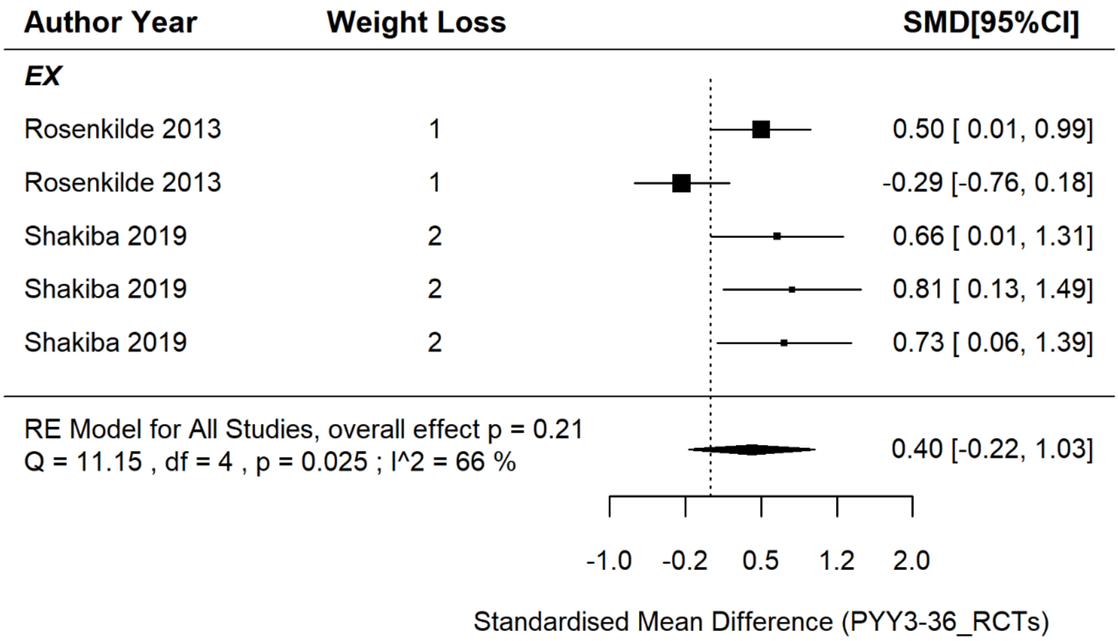

Figure S7. $PYY_{3\text{-}36}$ changes after weight loss from RCTs. N = 2 studies with 5 EX intervention groups. RE model: random effects model. The weight loss column is defined as follows.1: less than 5% weight loss and 2: 5-10% weight loss. The size of the squares in the figure is proportional to the weight of each study in the meta-analysis.

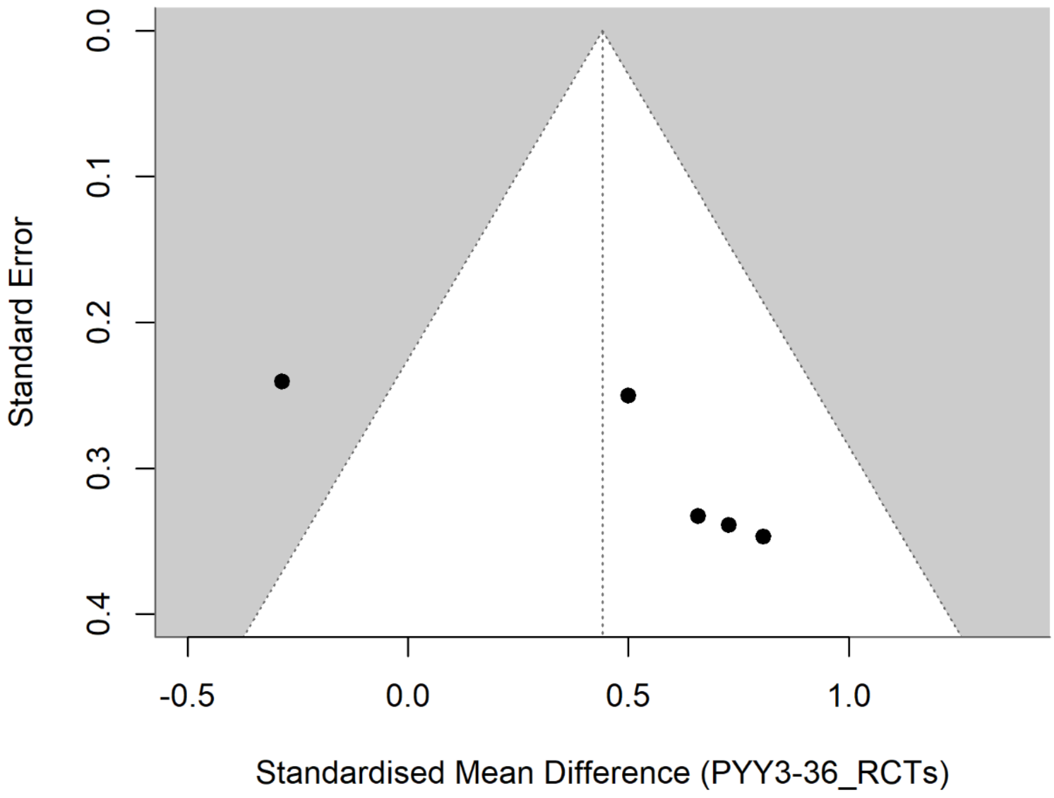

Figure S8. Funnel plot of PYY_{3-36} changes after weight loss from RCTs.

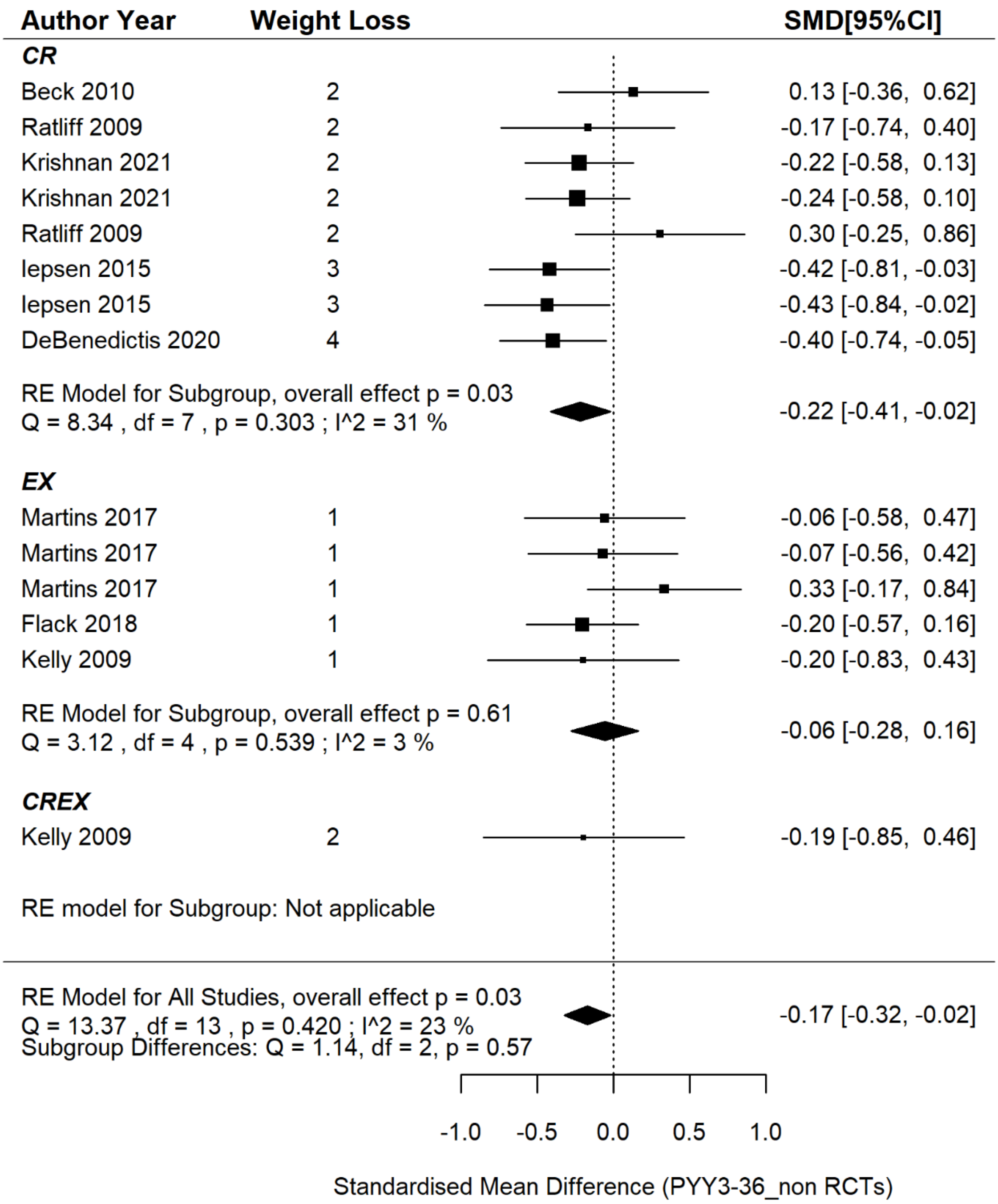

Figure S9. $PYY_{3\text{-}36}$ changes after weight loss from RCTs. N = 8 studies with 14 intervention groups (8 CR; 5 EX; 1 CREX). RE model: random effects model. The weight loss column is defined as follows.1: less than 5% weight loss; 2: 5-10% weight loss; 3: 10-15% weight loss and 4: 15-20% weight loss. The size of the squares in the figure is proportional to the weight of each study in the meta-analysis.

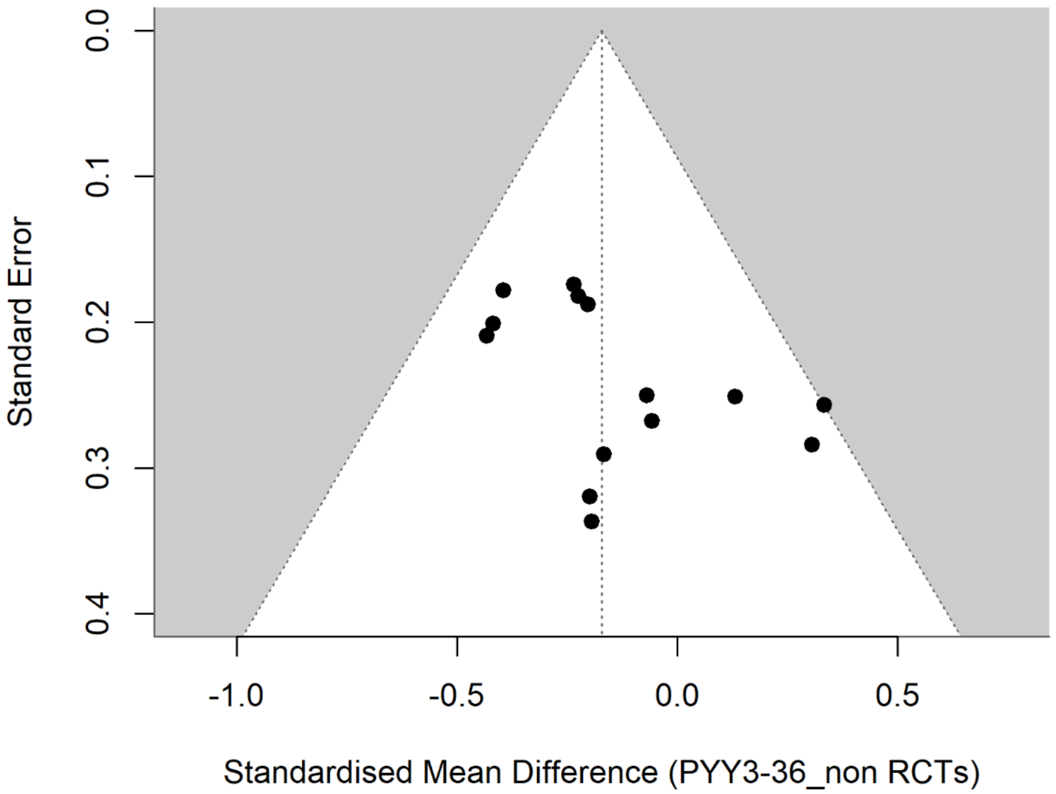

Figure S10. Funnel plot of PYY_{3-36} changes after weight loss from non-RCTs.

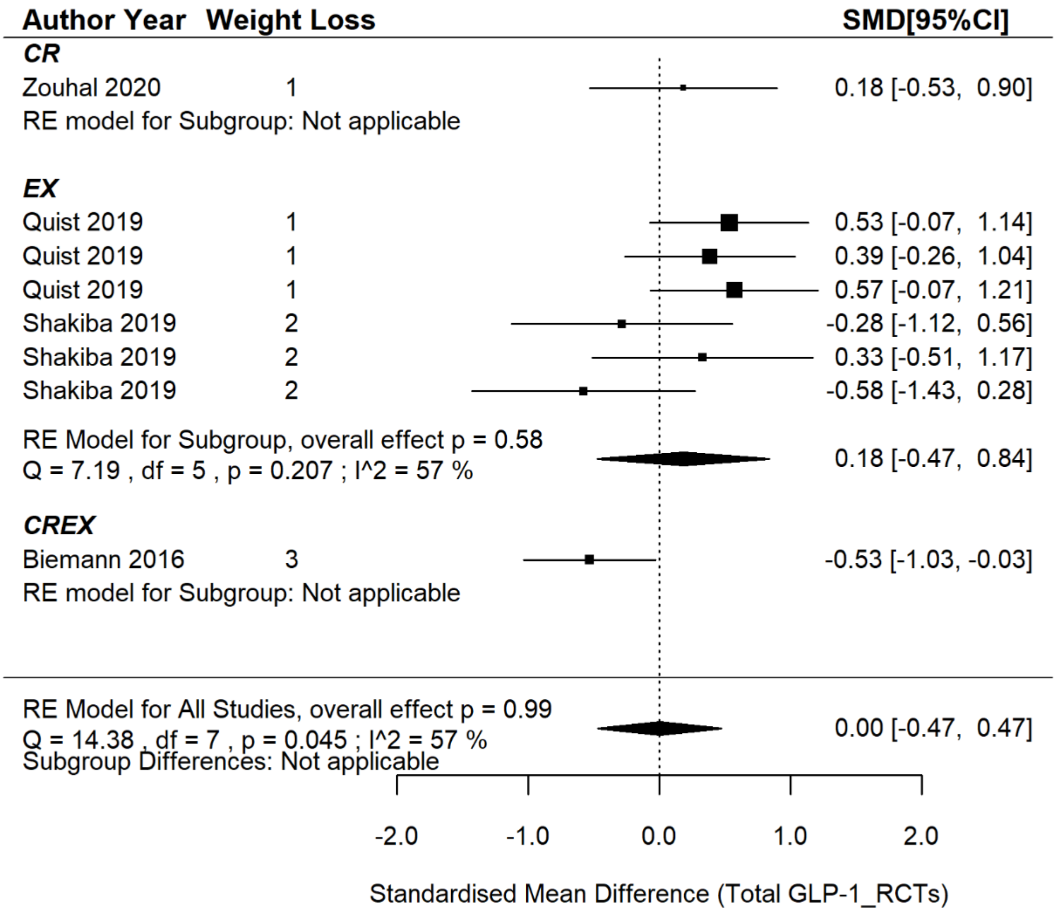

Figure S11. Total GLP-1 changes after weight loss from RCTs. N = 4 studies with 8 intervention groups (1 CR; 6 EX; 1 CREX). RE model: random effects model. The weight loss column is defined as follows.1: less than 5% weight loss; 2: 5-10% weight loss, and 3: 10-15% weight loss. The size of the squares in the figure is proportional to the weight of each study in the meta-analysis.

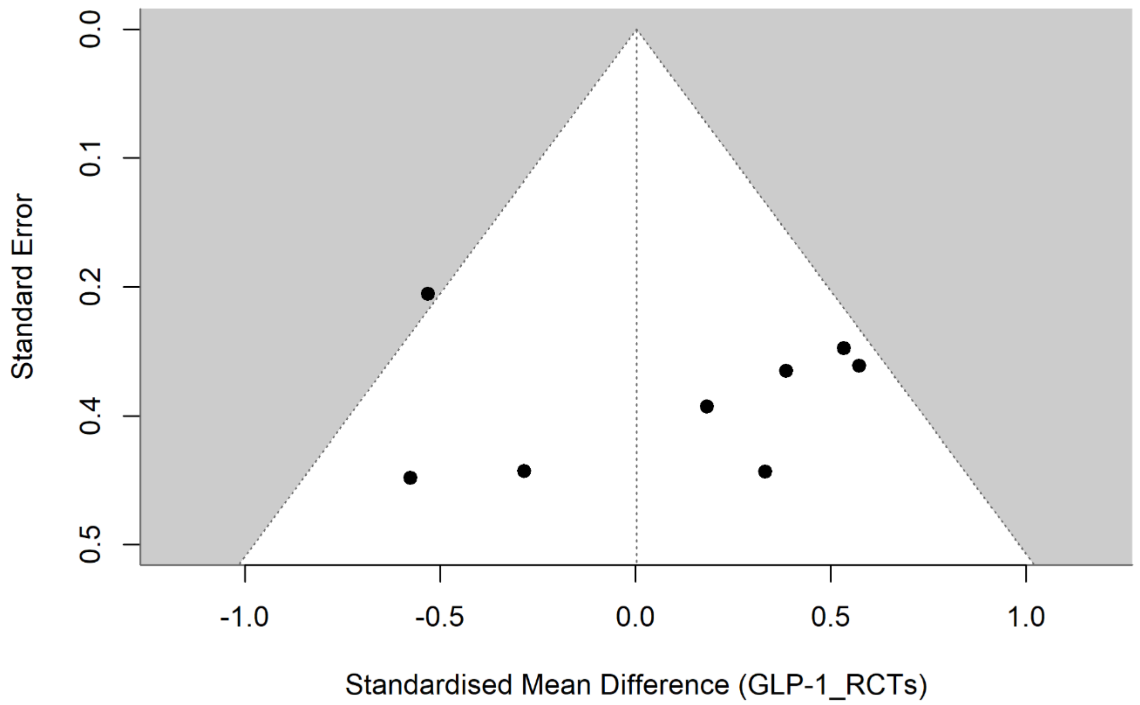

Figure S12. Funnel plot of total GLP-1 changes after weight loss from RCTs.

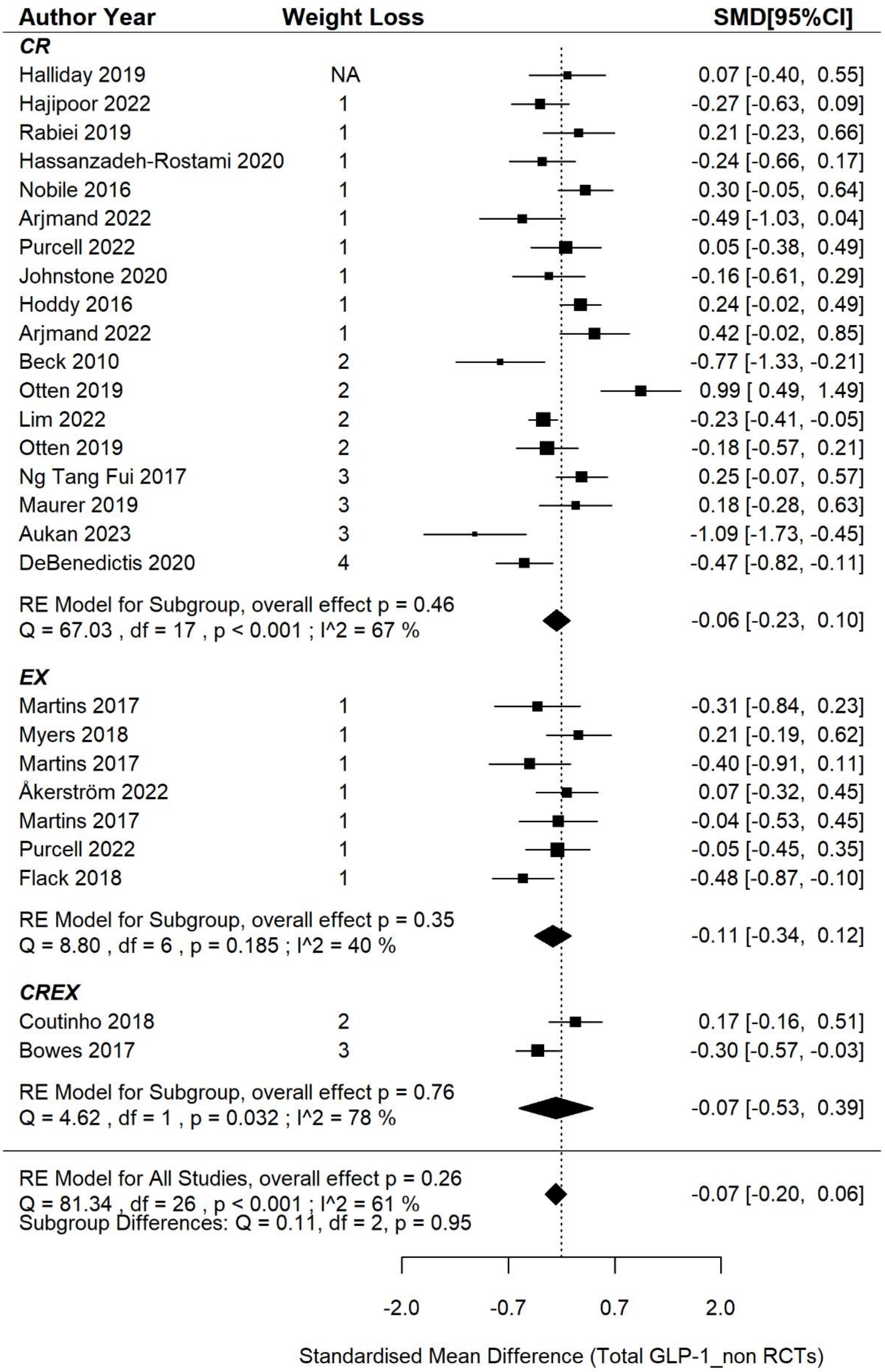

Figure S13. Total GLP-1 changes after weight loss from non-RCTs. N = 22 studies with 27 intervention groups (18 CR, 7 EX; 2 CREX). RE model: random effects model. The weight loss column is defined as follows.1: less than 5% weight loss; 2: 5-10% weight loss, 3: 10-15% weight loss, and 4: 15-20% weight loss. NA indicates that studies reported significant weight loss without providing exact numbers. The size of the squares in the figure is proportional to the weight of each study in the meta-analysis.

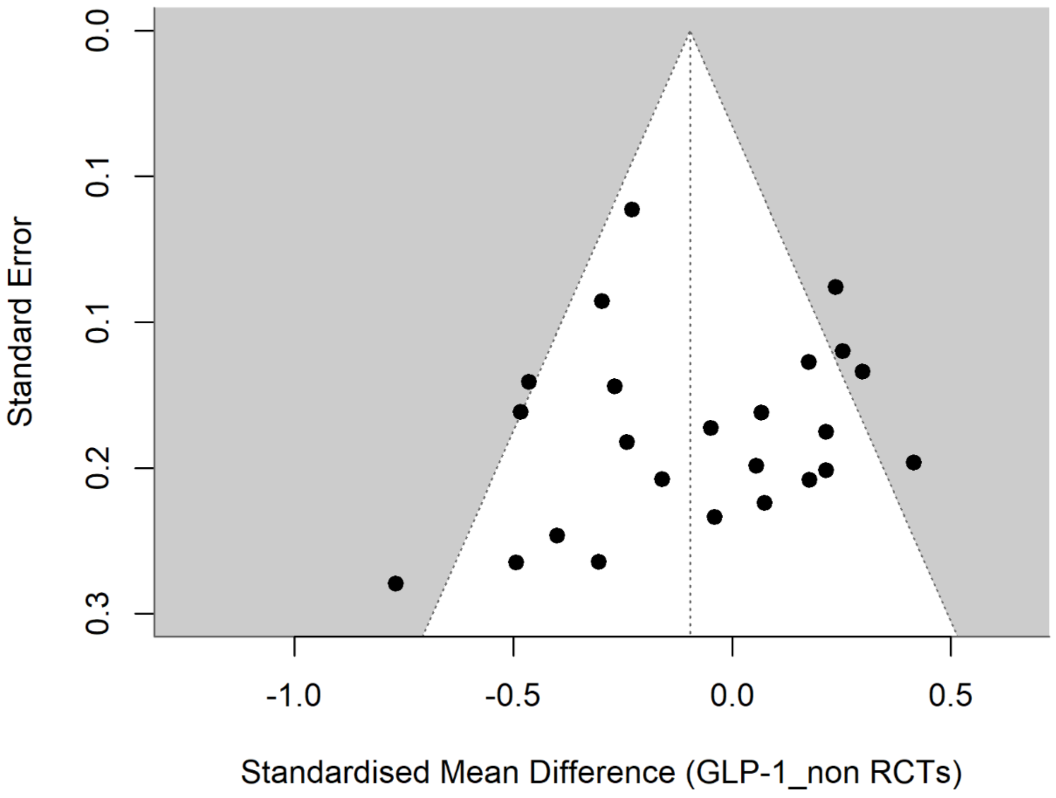

Figure S14. Funnel plot of total GLP-1 changes after weight loss from non-RCTs.

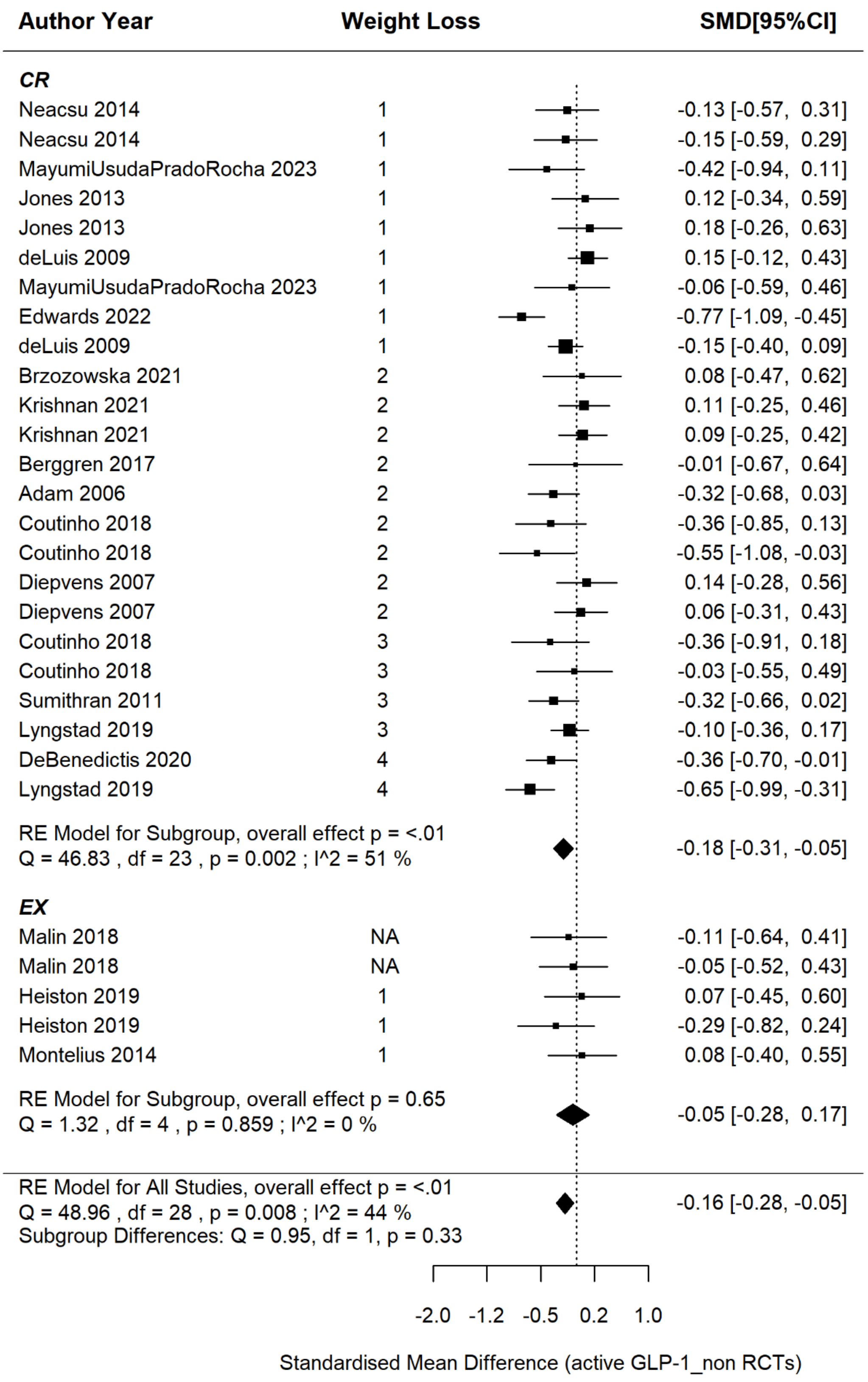

Figure S15. Active GLP-1 changes after weight loss from non-RCTs. N = 18 studies with 29 intervention groups (24 CR and 5 EX groups). RE model: random effects model. The weight loss column is defined as follows.1: less than 5% weight loss; 2: 5-10% weight loss, 3: 10-15% weight loss, and 4: 15-20% weight loss. NA indicates that studies reported significant weight loss without providing exact numbers. The size of the squares in the figure is proportional to the weight of each study in the meta-analysis.

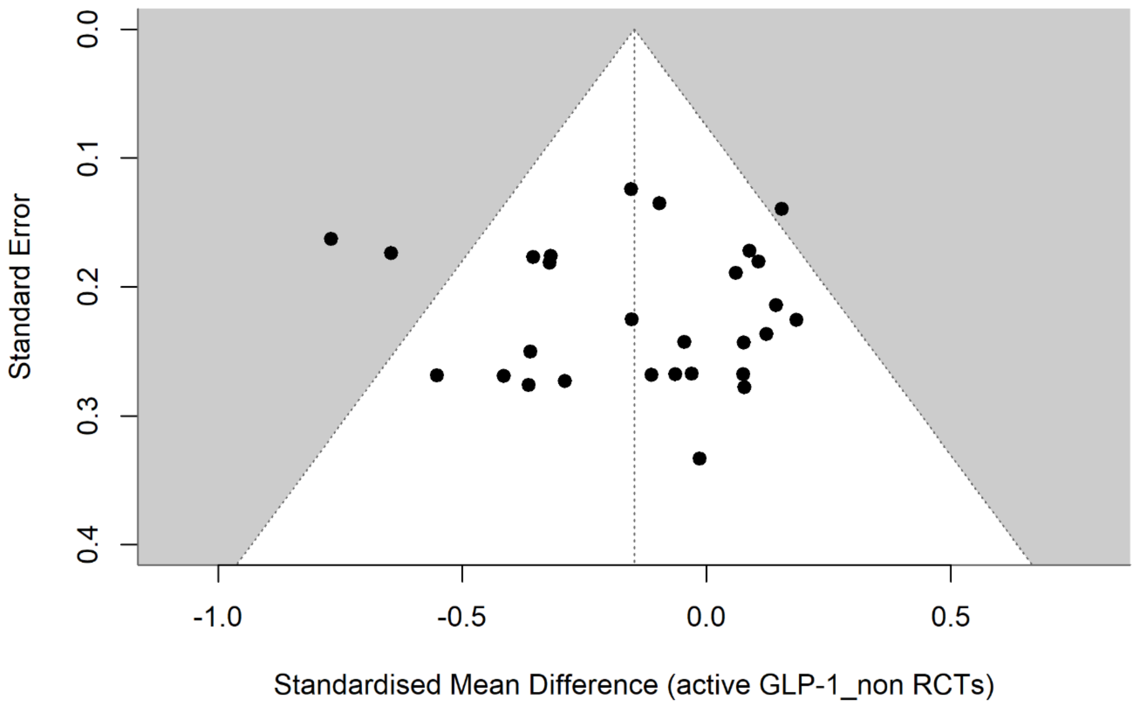

Figure S16. Funnel plot of active GLP-1 changes after weight loss from non-RCTs.

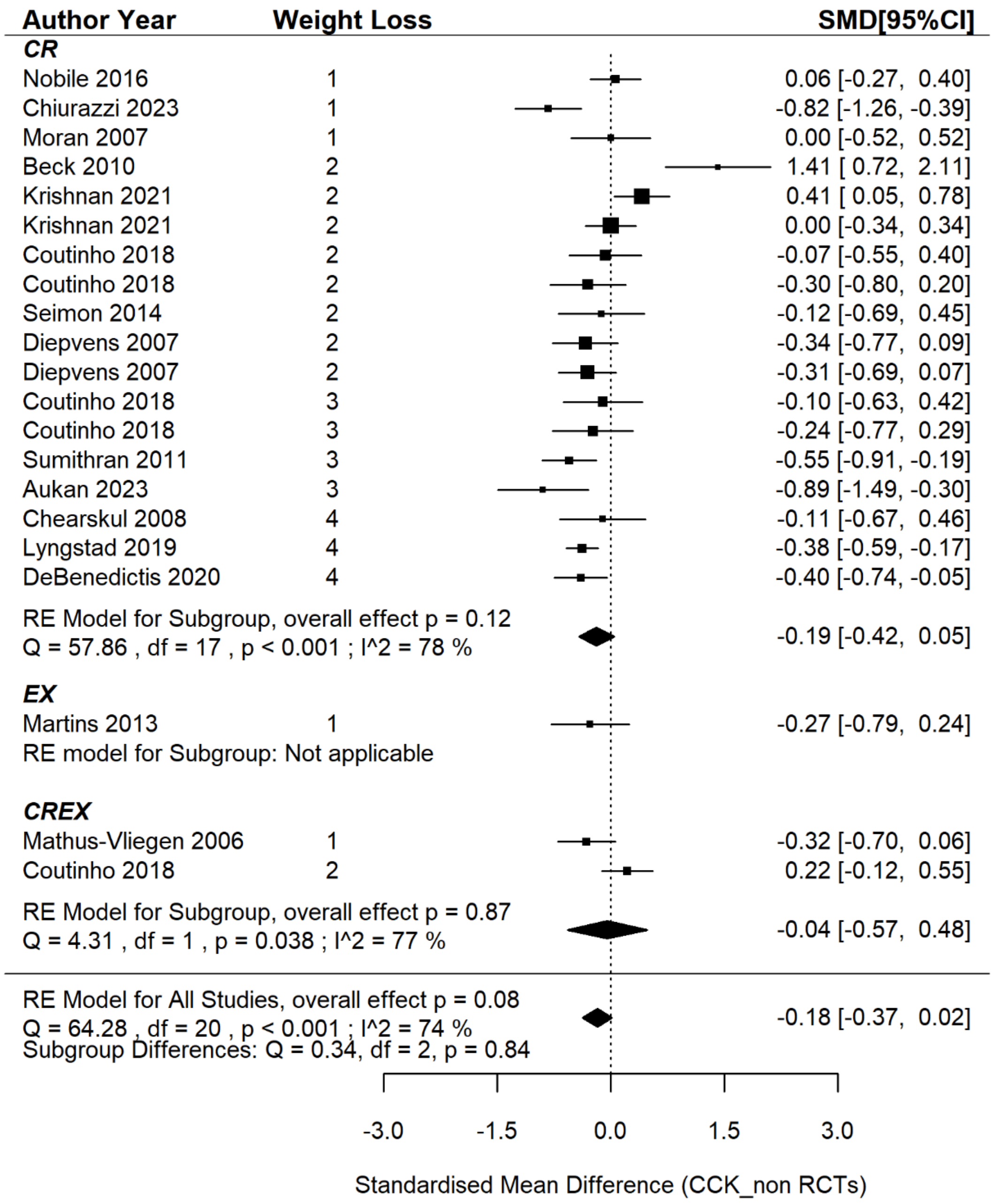

Figure S17. CCK changes after weight loss from non-RCTs. N = 17 studies with 21 intervention groups (18 CR, 1 EX and 2 CREX groups). RE model: random effects model. The weight loss column is defined as follows.1: less than 5% weight loss; 2: 5-10% weight loss, 3: 10-15% weight loss, and 4: 15-20% weight loss. The size of the squares in the figure is proportional to the weight of each study in the meta-analysis.

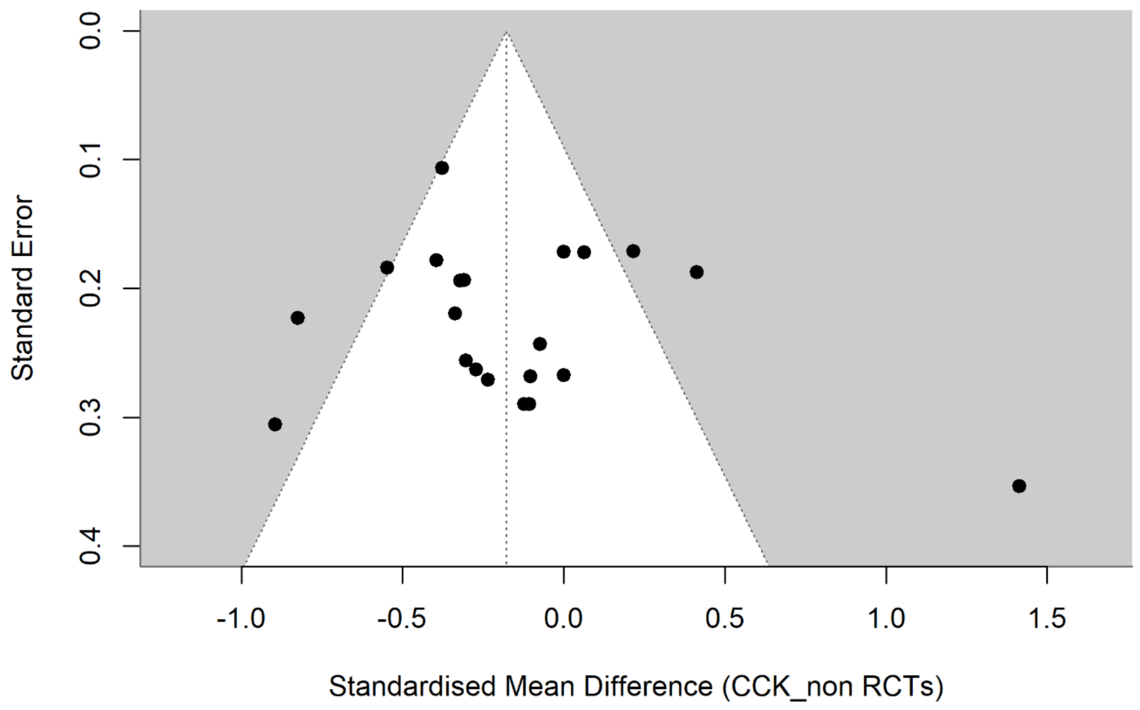

Figure S18. Funnel plot of CCK changes after weight loss from non-RCTs.

Reference Number	D1	D2	D3	D4	D5	Overall
44	+	+	+	+	!	+
45	+	+	+	+	+	+
46	!	+	+	+	!	+
47	+	+	+	+	!	+
48	!	+	+	+	!	+
49	+	+	+	+	!	+
50	+	+	+	+	!	+
51	+	+	+	+	+	+
52	+	+	+	+	+	+
53	+	+	!	+	+	!
54	!	+	+	+	!	!
55	+	!	!	-	!	-
56	+	+	+	+	!	+
57	+	+	+	+	+	+
58	+	+	+	+	!	+
59	!	!	!	+	!	!
60	+	!	+	+	+	+
61	!	+	+	!	!	!
62	!	!	!	+	+	!

+ Low risk

! Some concerns

- High risk

D1: Randomisation process

D2: Deviations from the intended interventions

D3: Missing outcome data

D4: Measurement of the outcome

D5: Selection of the reported result

Figure S19. Risk bias assessment of RCTs.

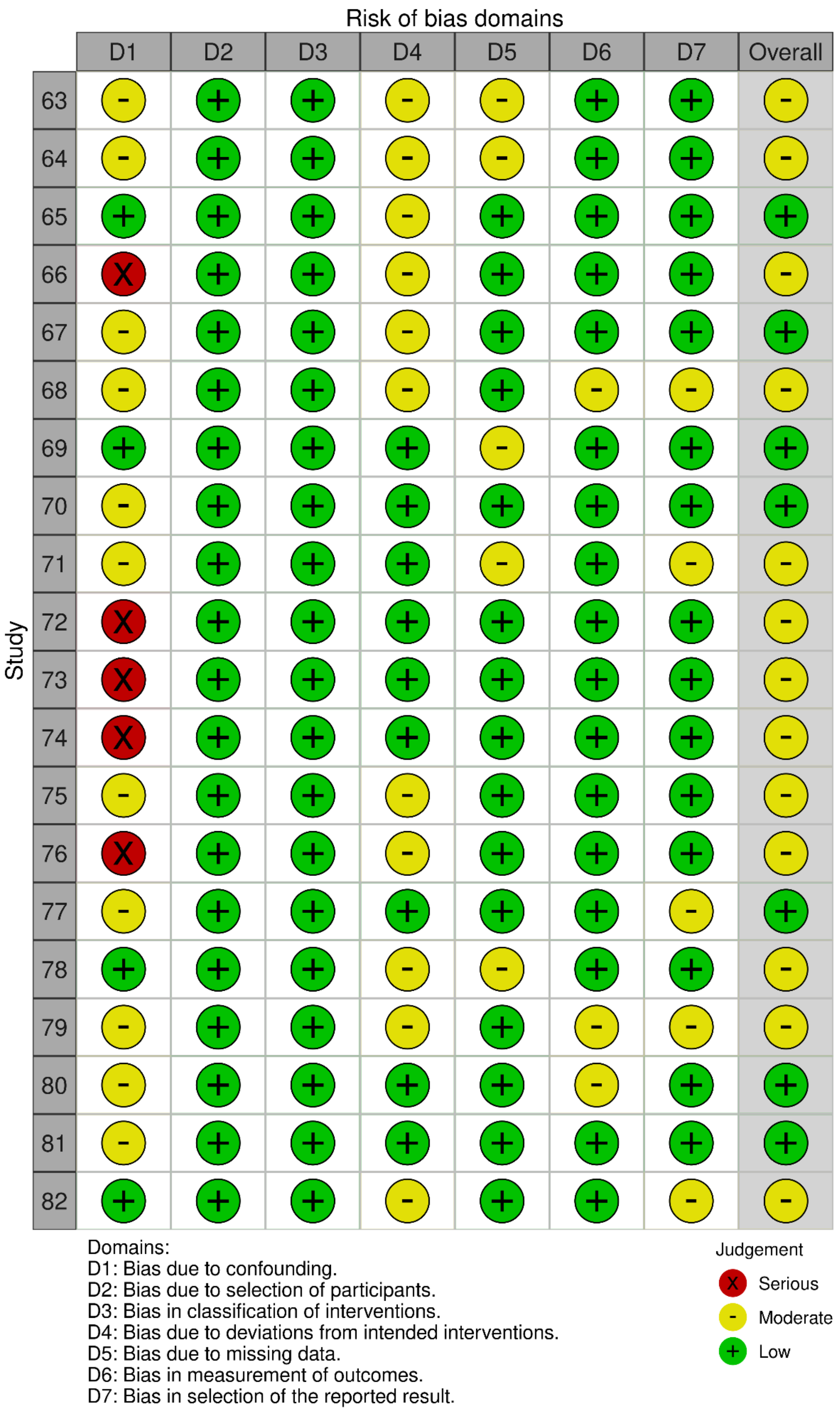

Figure S20. Risk bias assessment of non-RCTs (continued). The study number aligns with the reference number.

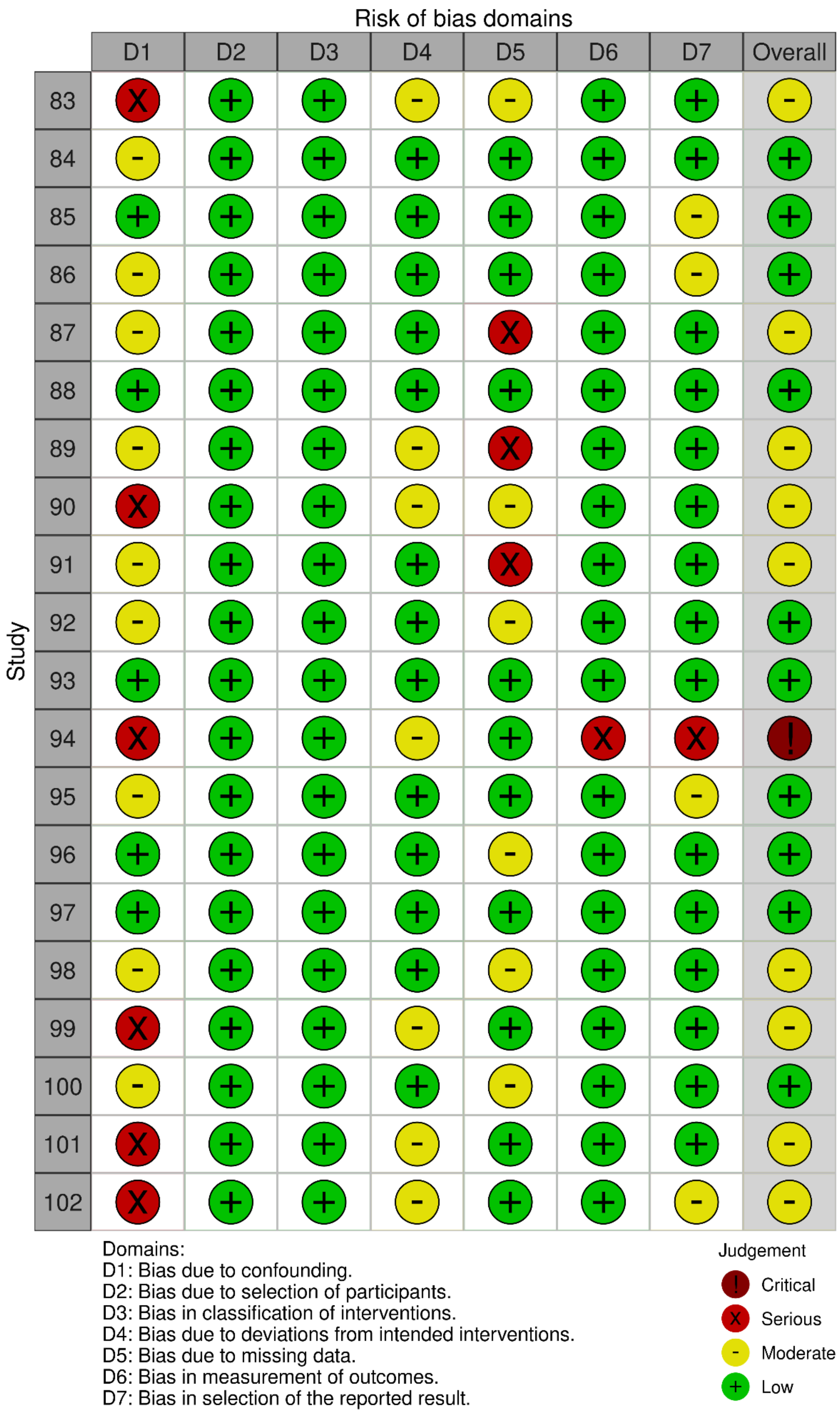

Figure S20. Risk bias assessment of non-RCTs (continued). The study number aligns with the reference number.

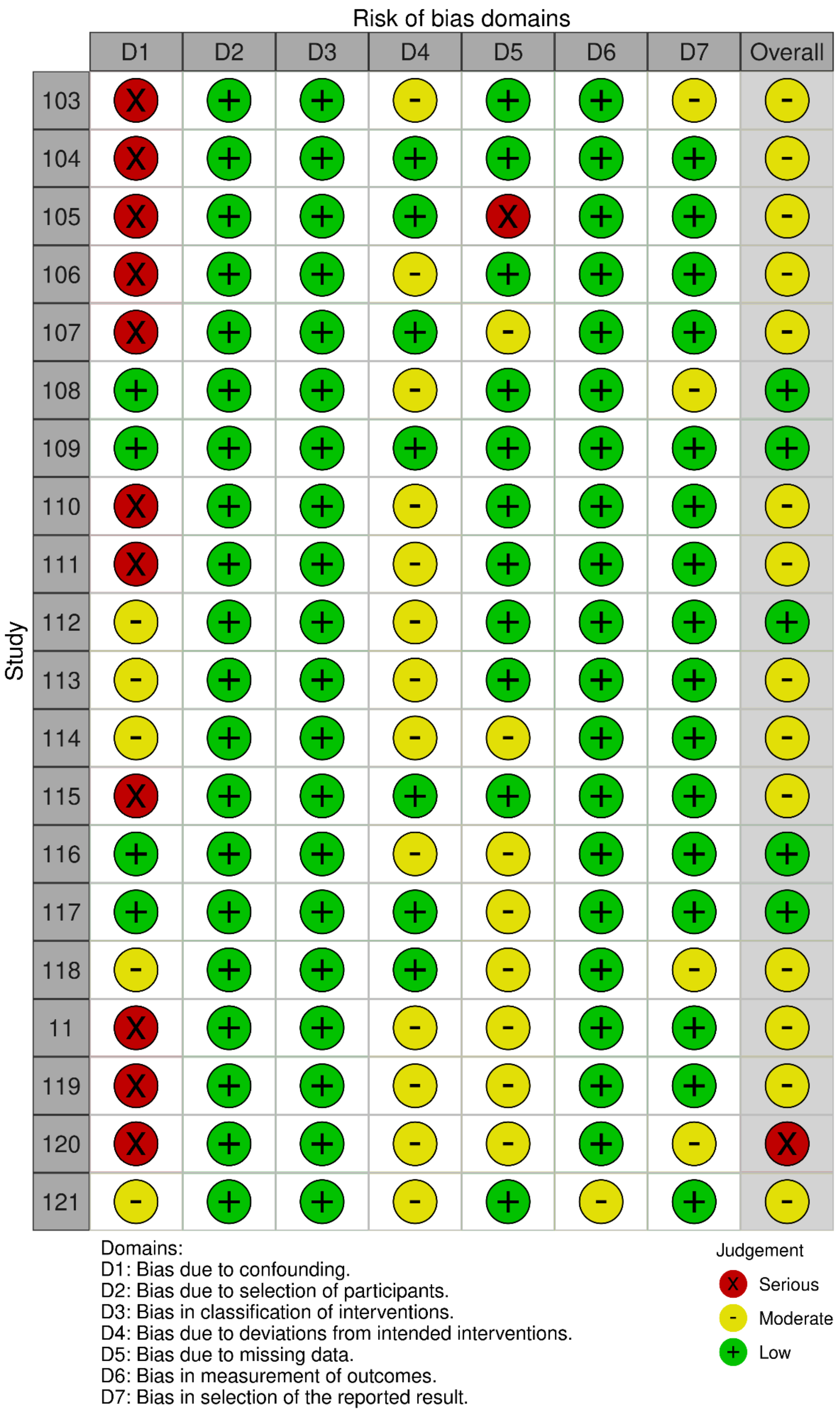

Figure S20. Risk bias assessment of non-RCTs (continued). The study number aligns with the reference number.

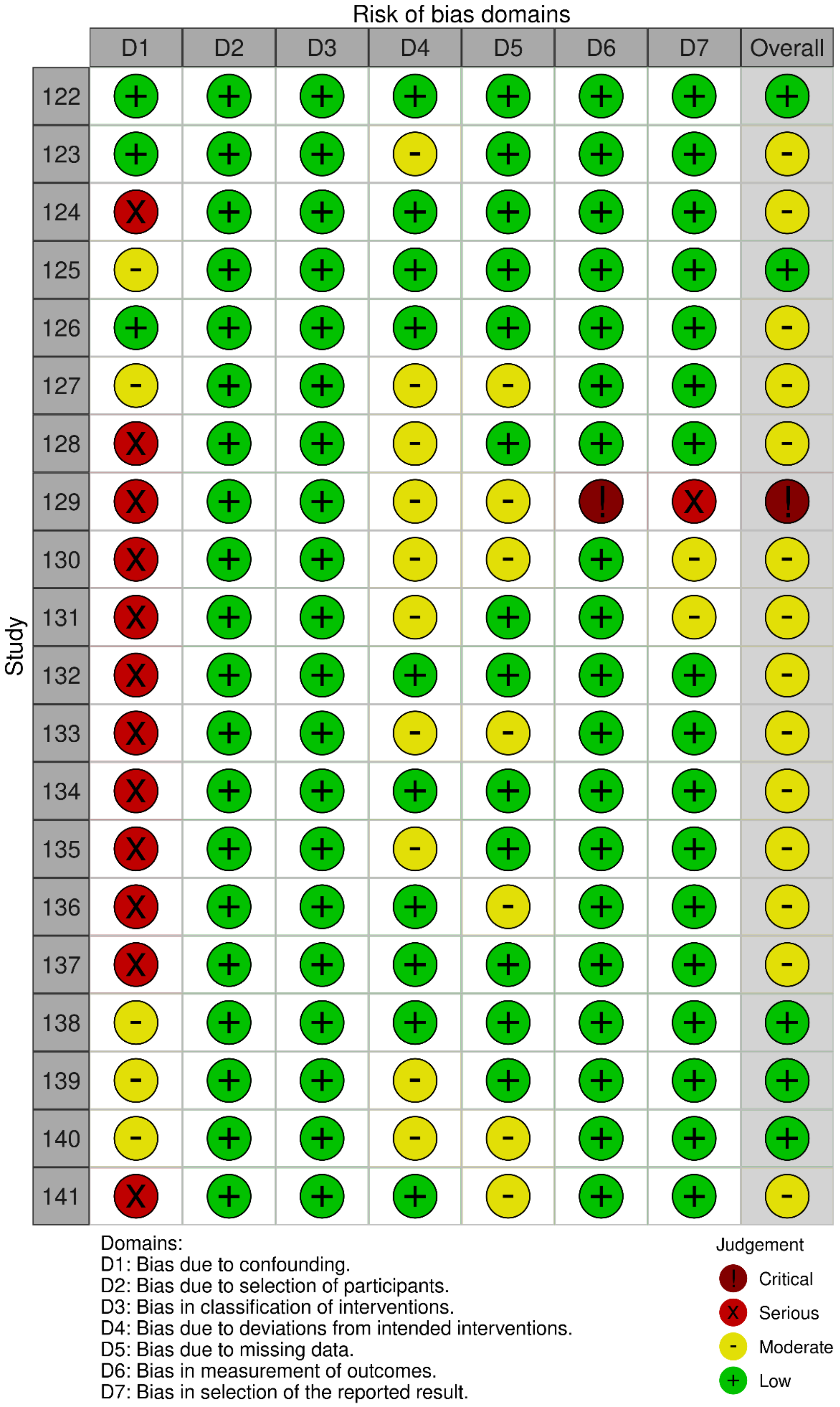

Figure S20. Risk bias assessment of non-RCTs (continued). The study number aligns with the reference number.

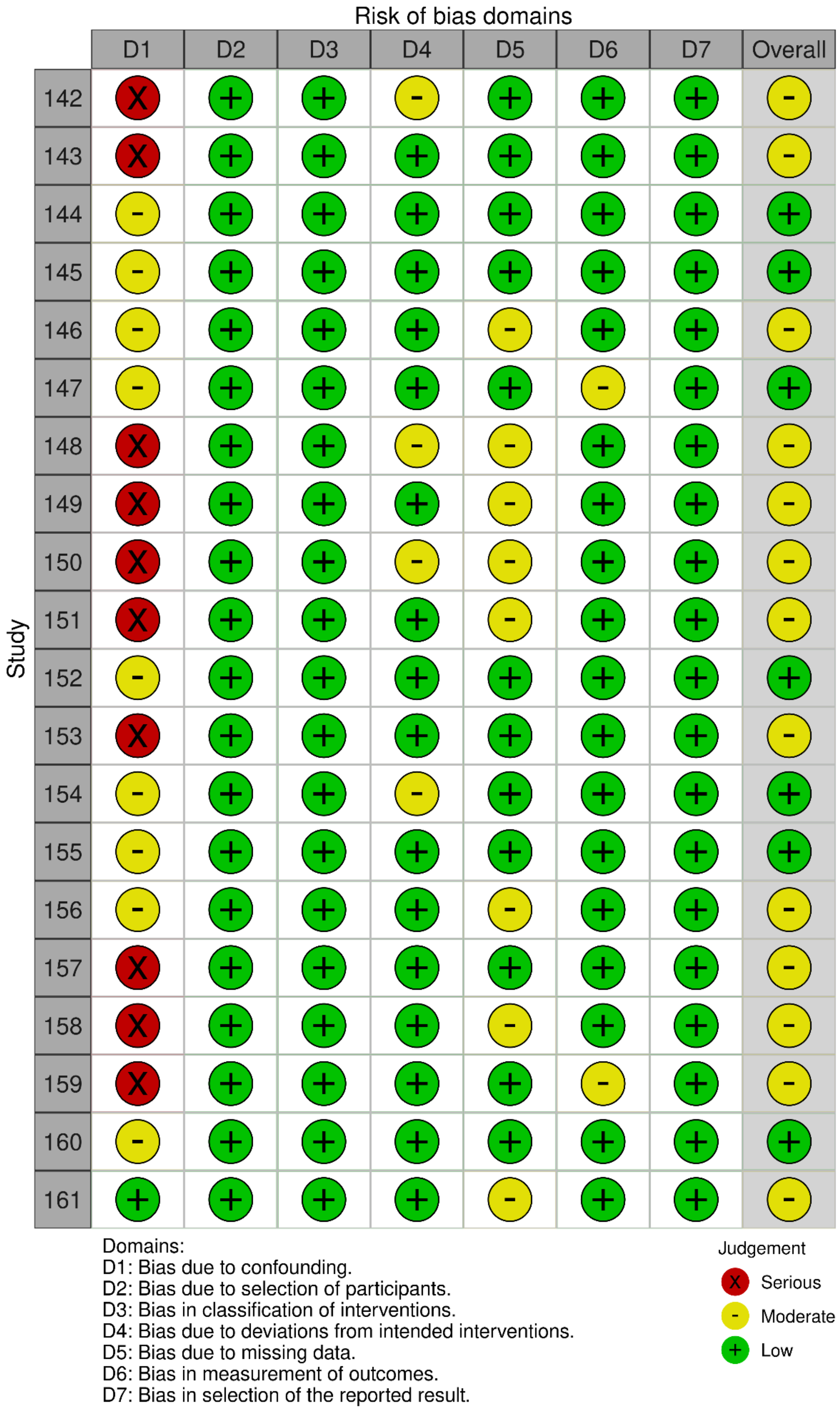

Figure S20. Risk bias assessment of non-RCTs (continued). The study number aligns with the reference number.

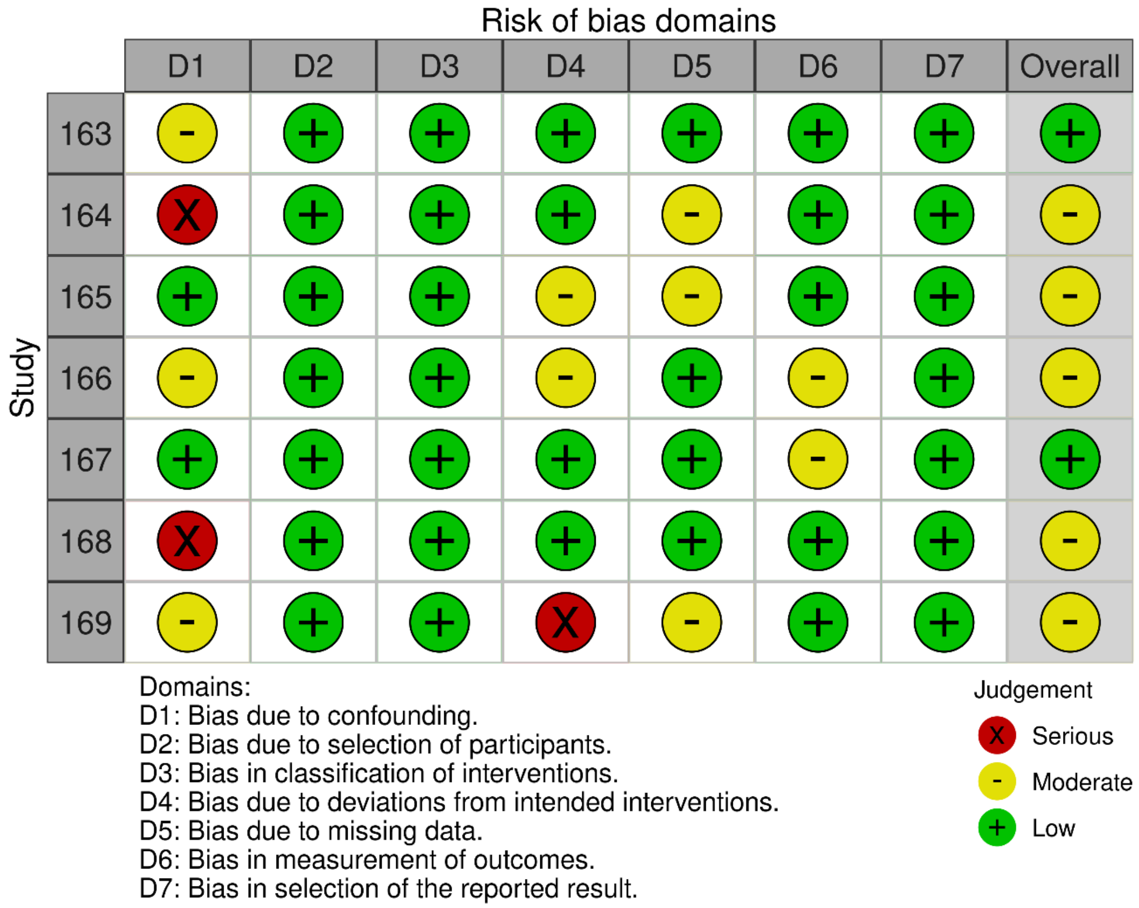

Figure S20. Risk bias assessment of non-RCTs. The study number aligns with the reference number.
