# Supplementary material for: Fasting appetite-related gut hormone responses after weight loss induced by calorie restriction, exercise, or both in people with overweight or obesity: a meta‐analysis
Source: Int J Obes (Lond). 2025 Feb 10;49(5):776–92. doi: 10.1038/s41366-025-01726-4 (PMC12095072; doi:10.1038/s41366-025-01726-4)
Supplement: Supplementary file 4 — Supplementary Table 1 [file 41366_2025_1726_MOESM4_ESM.pdf]

RCTs

Reference number	Author	Year	RCT	Intervention	Sex	n.int	n.c	Protocols	Duration (weeks)	Frequency (days/week)	bw.int (%)	bw.con (%)	Ghr. int	Ghr. con	AG. int	AG. con	PYY. int	PYY. con	PYY3-36.int	PYY3-36.con	GLP1 .int	GLP1. con	CCK. int	CCK. con
44	Barazzoni	2021	Yes	CR	F/M	11	9	-1000kcal/d + $P_{constant}$	16	7	10.20	-0.92	↑	→	→	→								
45	Biemann	2016	Yes	CREX	M	33	30	telemonitored lifestyle-induced weight loss program	26	7	13.80	-0.83									↓	→		
46	Guelfi	2013	Yes	EX	M	12	8	MICT at 75% HR_{max}	12	3	1.96	-0.11			→	→	→	→						
47	Rostamzadeh	2022	Yes	EX	M	15	15	4 sets of 8 reps with 80% of 1RM	24	3	11.29	-1.04			↓	→	↑	→						
48	Mager	2008	Yes	CR	F/M	28	18	-500 kJ/d + <30% EI fat	33	7	4.96	0.23	→	↓										
49	Mason	2015	Yes	EX	F	106	80	45min MICT at 70-85% of HRmax	52	5	2.75	0.95	→*	→										
				CR	F	105		1200–2000 kcal/d + <30% EI Fat	52	7	10.12	0.95	→*	→										
				CREX	F	108		CR + MICT	52	7(CR)/5(MICT)	13.08	0.95	↑*	→										
50	Quist	2019	Yes	EX	F/M	22	16	BIKE at 54% VO_{2peak} with EE 320(w) and 420 (m)kcal/d	26	5	2.00	-2.25			→*	→	→*	→			→*	→	→	→
				EX	F/M	33		MICT at 49% VO_{2peak} with EE 320(w) and 420 (m)kcal/d	26	5	0.45	-2.25			→*	→	→*	→			→*	→	→	→
				EX	F/M	25		MICT at 66% VO_{2peak} with EE 320(w) and 420 (m)kcal/d	26	5	2.06	-2.25			→*	→	→*	→			↑*	→	→	→
51	Rosenkilde	2013	Yes	EX	M	18	17	30min MICT at 66% VO2max	12	7	3.76	-0.11	→	→					→	→				
				EX	M	18		55min MICT at 67% VO2max	12	7	2.74	-0.11	→	→					↑*	→				
52	Shakiba	2019	Yes	EX	M	11	11	3 *(10min running at 80-90% HRmax + 5min walking)	12	3	4.96	-0.16			↓	→			↑	→	→	→		
				EX	M	11		4 sets of 8 reps with 80% of 1RM	12	3	6.20	-0.16			↓	→			↑	→	→	→		
				EX	M	11		1 aer + 1 re	12	3	5.55	-0.16			↓	→			↑	→	→	→		
53	Sheikholeslami-Vatani	2022	Yes	EX	M	12	12	40min HIIT at 90% VO_{2peak}	8	3	4.46	-0.06			→	→	↑	→						
54	Zouhal	2020	Yes	CR	M	15	15	-20%EI + 15-16h fasting/d	4	7	3.37	0.30			→	→	↓	→			↓	→	↓	→
55	Kang	2018	Yes	EX	F	13	13	50min (walking + re)	12	5	7.98	0.65	↑	↓										
56	Redman	2010	Yes	CR	F/M	11	11	-25% ER	26	7	10.40	-1.1	↑	→										
				CREX	F/M	12		12.5% CR + 12.5% aer	26	7	10.00	-1.1	↑	→										
				CR	F/M	9		890kcal/d	26	7	13.90	-1.1	↑	→										
57	Foster-Schubert	2005	Yes	EX	F	87	86	45min MICT at 60-75% HR_{max}	52	5	1.71	-0.12	↑	→										
58	Sun	2021	Yes	EX	F	13	11	2.5minHIIT at 86.5% VO2max	4	5	4.05	3.87	→	→										
59	Ahmadi	2019	Yes	EX	M	15	15	50-60min MICT at 60-85% of HRmax	8	4	3.59	3.59	↑	→										
60	Valinejad	2022	Yes	EX	M	8	8	40-60min MICT 60–70% of the HRmax	6	3	9.16	6.46			↑	↑								
			Yes	EX	M	8	8	3–5 series with 35 s of rest between each re exercise	6	3	7.82	6.46			↑	↑								
61	Hu	2022	Yes	EX	F	26	25	combined MICT and HIIT	4	5	4.50	3.56	→	→										
62	Mohammad	2022	Yes	EX	M	15	15	80 -90 min re	8	3	6.20	0.70	→	→										

n.int: sample size of intervention group; n.c: sample size of control group; bw.int(%): body weight loss percentage in intervention group; bw.con(%): body weight loss percentage in control group; * compared with control group at post-intervention time point. Pconstant: keep protein intake constant the same as pre-intervention; MICT: moderate-intensity continous training; HR: heart rate; EI: energy intake; CR: calorie restriction; BIKE: using bike as active commuting; (w) and (m): woman and man; aer: aerobic training; re: resistance trainning; LC: low carbohydrate diet; HIIT: high-intensity interval training

non- RCTs

Reference number	Author	Year	Intervention	Sex	n.int	Protocols	Duration (weeks)	Frequency (days/week)	bw(%)	Ghr	AG	PYY	PYY 3-36	GLP1	aGLP1	CCK
63	Adam	2006	CR	F/M	32	2540 kJ/d	6	7	8.07						→	
64	Berggren	2017	CR	F	9	Very Low Calorie Diet (VLCD) regimen	4	7	6.74						→	
65	Brzozowska	2021	CR	F/M	13	VLCD	52	7	5.21			→			→	
66	Cameron	2010	CR	F/M	8	-2931 kJ/d (3meals+3snack)	8	7	4.03	↑		↓				
	Cameron	2010	CR	F/M	8	-2931 kJ/d (3meals)	8	7	5.25	↑		↓				
67	Chearskul	2008	CR	M	12	1800 kJ/d	8	7	15.34							→
68	Cooper	2013	CREX	F/M	233	decrease EI + increase EE	26	7	7.84	↑						
69	Coutinho	2018	CR	F/M	14	550(w) and 660(m) kcal/d	12	3	12.97		↑	→			→	→
	Coutinho	2018	CR	F/M	14	low calorie diet (LCD)	12	7	12.10		→	→			↓	→
70	Coutinho	2018	CR	F/M	17	550(w) and 660(m) kcal/d	4	7	9.21		→	↓			→	→
	Coutinho	2018	CR	F/M	16	1200(w) and 1500(m) kcal/d	8	7	9.36		→	↓			→	→
71	Crujeiras	2010	CR	F/M	104	-(500-600) kcal/d (-30%ER)	8	7	5.36	→						
72	Cummings	2002	CR	F/M	13	1000kcal/d + HP	12	7	17.43	↑						
73	DeBenedictis	2020	CR	F/M	34	550(w) or 660(m) kcal/d	12	7	16.99		↑	→	→	→	→	→
74	deLuis	2009	CR	F/M	52	1507 kcal/day + LC	12	7	3.62						→	
	deLuis	2009	CR	F/M	66	1500 kcal/day + LF	12	7	4.92						↓	
75	deLuis	2008	CR	F/M	20	1520kcal/d	12	7	8.92		→					
76	Diepvens	2007	CR	F	22	2.1MJ/d	6	7	9.59		→				→	→
	Diepvens	2007	CR	F	28	2.1MJ/d	6	7	9.75		↓				→	→
77	Dinu	2020	CR	F/M	53	1400 (w) and 1600 kcal/d (m) + MD	12	7	2.11	→						
	Dinu	2020	CR	F/M	54	1400 (w) and 1600 kcal/d (m) + VD	12	7	2.29	→						
78	Doucet	2004	CR	M	15	-800kcal/d	0.5	7	1.46	→						
79	Elerian	2020	CREX	F/M	12	1200–1800 kcal/d + 50min re (3 times/wk)	14	7/3	10.78	→						
80	Figueroa-Vega	2020	CR	F/M	52	-580kcal/d	7	7	4.55		→					
81	Gilbert	2011	CR	F	13	-600kcal/d + Ca	26	7	9.10	→						
	Gilbert	2011	CR	F	12	-600kcal/d	26	7	6.73	→						
82	Halliday	2019	CR	F/M	17	800 kcal/d + LC	6	7	NA	↑		→		→		
83	Hansen	2002	CREX	F	8	decrease EI + increase EE	26	7	5.23	↑						
84	Hayes	2007	CR	F/M	20	from 2079.3(196.2) to 1322.8(105.9) + LC	12	7	5.56	↑						
85	Heinonen	2009	CR	F/M	35	800 kcal/d	8	7	14.55	↑						
86	Heiston	2019	EX	F/M	14	60min cycling at 70% HRpeak	2	7	0.32		→				→	
86	Heiston	2019	EX	F/M	14	60min of HIIT at 90%and 50% HRpeak	2	7	0.90		→				→	

87	Hoddy	2016	CR	F/M	59	25% ER on fasting day	8		4.17	→		→		→		
88	Horner	2021	EX	M	15	alter (30min 50%VO2max MICT + HIIT)	4	5	0.94	→						
89	Jakubowicz	2013	CR	F	38	1400kal/d +HB	12	7	10.06	↓						
	Jakubowicz	2013	CR	F	36	1400kal/d + HD	12	7	4.13	↓						
90	Johnstone	2020	CR	F/M	19	100% RMR	3	7	3.86	↑		→		→		
91	Jones	2013	CR	F/M	18	-500kcal/d	12	7	2.62		→	→			→	
	Jones	2013	CR	F/M	20	-500kcal/d	12	7	3.52		→	→			→	
92	Kelly	2009	EX	F/M	10	50-60 min MICT at 75%VO2max	12	5	3.01				→			
	Kelly	2009	CREX	F/M	9	-700kcal/d + 50-60min MICT at 75%VO2max	12	7/5	8.43				→			
93	Krishnan	2021	CR	F/M	34	-500 kcal/d + low dairy	12	7	6.73		↑		→		→	→
	Krishnan	2021	CR	F/M	31	-500 kcal/d + adequate dairy	12	7	6.70		↑		→		→	→
94	Liu	2021	CREX	F/M	47	daily EI ≤125.7 kJ/kg + 30min brisk walking	NA	7/5	1.95			→				
95	Lopes	2013	CR	F/M	9	-(500-1000)kcal/d	12	7	5.48		→					
	Lopes	2013	CREX	F/M	9	-(500-1000)kcal/d + 40min cycling at 70% of HR reserve	12	7/3	5.18		↓					
96	Malin	2018	EX	F/M	17	MICT at 70% HRpeak	2	7	NA						→	
	Malin	2018	EX	F/M	14	alternating 3min(90%+ 50% HRpeak)	2	7	NA						→	
97	Malin	2020	CR	F	13	-800kcal/d	2	7	2.54		→	→				
	Malin	2020	CREX	F	13	-800kcal/d + 60min HIIT at 90% HRpeak	2	7	1.54		→	→				
98	Martins	2017	EX	F/M	14	MICT at 70% of HRmax to 250kcalEE	12	3	0.79		→		→	→		
	Martins	2017	EX	F/M	16	HIIT at 85%-90% of HRmax to 250kcalEE	12	3	1.33		→		→	→		
	Martins	2017	EX	F/M	16	HIIT at 85%-90% of HRmax to 125kcalEE	12	3	1.87		→		→	→		
99	Maurer	2019	CR	F/M	19	800kcal/d (8wks) + 1500kcal/d(12wks)	12	7	12.46					→		
100	Martins	2013	EX	F/M	15	cycling at 75% HRmax to provide 500kcal ED	12	5	3.64							→
101	Mathus-Vliegen	2006	CREX	F/M	28	-600kcal/d (1200kcal/d EI minimal) + PA	4	7	2.13							→
102	Mohammed	2014	CREX	F/M	40	1500 kcal/d + 45min walking	39	7	4.33	→						
103	Montelius	2014	EX	F	17	30min low intensity exercise	12	7	4.36	→					→	
104	Moran	2007	CR	F	14	-30% EI	8	7	4.95	→		→				→
105	Moran	2005	CR	F/M	57	12wk 6081KJ/d + 4wk 7346KJ/d	16	7	9.49	↑						
106	Morante	2020	CREX	F/M	18	-1000kcal/d + 150min aer /wk	12	7	10.07	→						
107	Morpurgo	2003	CREX	F/M	10	-500kcal/d + 30min bike (30-45% HRmax) + 50-70min brisk walking	3	7/5	5.65	→						
108	Näätänen	2021	CR	F/M	42	600kcal/d EI + HSF	7	7	12.54	↑		↓				
	Näätänen	2021	CR	F/M	40	600kcal/d EI + LSF	7	7	12.68	↑		↓				
109	Neacsu	2014	CR	M	20	8.7MJ/d + Soy-HPWL	2	7	2.20	→		→			→	
	Neacsu	2014	CR	M		8.7MJ/d + Meat-HPWL	2	7	2.07	→		→			→	

110	Neseliler	2019	CREX	F/M	24	1100-1400 kcal/d +30-40min brisk walking	12	7	6.19	↑	↑					
111	Nobile	2016	CR	F/M	34	-300kcal/d	12	7	2.63					↑		↑
112	Ozcan	2015	EX	F	20	60min 60% -70% HRmax	16	4	6.08	→						
	Ozcan	2015	EX	F	20	60min 10reps*3sets core re	16	4	5.89	→						
113	Ozkan	2009	CR	F/M	10	24 kcal/kg/d	12	7	3.04	↑						
114	Rashad	2019	CREX	F	60	-500kcal/d + 150min walking/wk	12	7	12.71	↑						
115	Ratliff	2009	CR	M	13	-2.1MJ/d + cholesterol	12	7	6.77	→			→			
	Ratliff	2009	CR	M	12	-2.1 MJ/d	12	7	6.05	→			→			
116	Romon	2006	CR	F	17	800 kcal/d	7	7	10.31	↑						
117	Seimon	2014	CR	M	12	-30% EI	12	7	9.43	→		→				→
118	Sofer	2013	CR	F/M	30	1300-1500 kcal/d + carbohydrate at dinner	12	7	NA	→						
	S. Sofer	2013	CR	F/M	33	1300-1500 kcal/d	12	7	NA	→						
11	Sumithran	2011	CR	F/M	34	500 -550 kcal/d	10	7	14.02		↑	↓			→	↓
119	Tremblay	2019	EX	F/M	24	high-re(70%) and mod-en intensity (-500kcal/d)	52	4	7.26	→						
119	Tremblay	2019	EX	F/M	24	mod-re (30%) and high-en (70%) intensity (-500kcal/d)	52	4	9.68	→						
119	Tremblay	2019	EX	F/M	30	mod-re (30%) and mod-en (30%) intensity (-500kcal/d)	52	4	7.30	→						
120	Zahorska-Markiewicz	2004	CREX	F	35	1000kcal/d + PA	12	7	9.02	↑						
121	Hassanzadeh-Rostami	2020	CR	F/M	23	-500 kcal/d	8	7	2.29	↓				→		
122	Blom	2009	CR	M	11	36% of ER	0.5	3	2.09	↑						
123	Catenacci	2016	CR	F/M	12	-400kcal/d	8	7	6.23	→						
	Catenacci	2016	CR	F/M	13	Alternate-Day Fasting	8	alternate	8.65	↑						
124	Kudiganti	2016	EX	F/M	28	30 min walk (5d/wk) + 2000kcal/d	16	5	1.44	→						
125	Jensen	2012	CR	F/M	42	> = -300 kcal/d; EI> 1200kcal/d	12	7	5.06	↓						
126	Hill	2013	CR	F	71	LED (1886 ± 430 to 1351 ± 345)kcal/d	26	7	8.63	↑		↑				
127	Huerta	2015	CR	F	22	-30% EE	10	7	6.15	→						
128	Iepsen	2015	CR	F/M	27	800kcal/d	8	7	12.11	↑			↓			
	Iepsen	2015	CR	F/M	25	800kcal/d	8	7	12.38	↑			↓			
129	Ippoliti	2008	CR	F/M	30	900kcal/d	6	7	3.55	↑						
130	Lien	2009	CREX	F/M	27	decrease EI + increase EE	26	7	6.19	↑		↓				
131	Pamuk	2018	CR	F	26	24 kcal/kg	12	7	5.02	↑						
132	Mohammadi-Sartang	2019	CR	F/M	43	-500kcal/d + 300 mg Ca	10	7	5.16	→						
133	Purcell	2014	CR	F/M	76	450-800kcal/d	12	7	15.15	↑						

133	Purcell	2014	CR	F/M	51	-(400-500)kcal/d	36	7	14.71	↑						
134	Ramel	2009	CR	F/M	69	-30% EE	8	7	5.02	↑						
135	Ravussin	2001	EX	M	14	extra 1000kcal/d EE on ergometer	13	9d on + 1d off	6.09	↑						
136	Sadeghian	2021	CR	F	30	500 kcal/day	8	7	2.89	→						
	Sadeghian	2021	CR	F	30	fast-mimicking diet	8	5d/m	1.37	↓						
137	Santosa	2007	CREX	F	35	-20% EI +10%EE	26	7	14.37	↑						
138	Rondanelli	2013	CR	F/M	45	-600kcal/d	8	7	NA	→						
139	Siebert	2021	CR	F/M	62	-80% EI on fasting day (3d/wk)	26	7	8.50	↑		→				
140	Wang	2018	CR	F/M	15	57–71% of EI	8	7	3.75	→						
141	Rondanelli	2022	CREX	F/M	12	(630–700 kcal/d + keto; 800–1500 kcal/d) + keto; 1500–2000 kcal/d + ae	12	7/3re+1h aer	11.84	↑						
142	Coutinho	2018	CREX	F/M	35	decrease EI + increase EE	104	7	8.29		→	→		→		→
143	Flack	2018	EX	F/M	29	combine 1500 and 300kcal/wk EE; 2 MICT (%HRR zones 1 and 2) 3 HIIT (%HRRzones 3 and 4)	12	5	2.34		↑		→	↓		
144	Myers	2018	EX	F	24	70% HRmax to 500 kcal EE	12	5	1.08		→	→		→		
145	Grangeiro	2020	CR	F	19	-700 kcal/d + 6 meals/d	12	7	3.29		→					
	Grangeiro	2020	CR	F	21	-700 kcal/d + 3 meals/d	12	7	3.87		↑					
146	Lim	2022	CR	F	121	3.9 MJ or 4.6 MJ/d (40% ER)	8	7	7.91			→		→		
147	Rabiei	2019	CR	F/M	20	-500kcal/d	12	7	2.14			→		→		
148	Fui	2017	CR	M	38	640 kcal/d	10	7	11.18	→		→		→		
149	Beck	2010	CR	F	16	-2MJ/d + fibre	12	7	5.15	→		↓	→	↓		↑
150	Buso	2021	CR	F/M	136	800 kcal/d	8	7	11.55	↑		↓				
151	Hainer	2008	CR	F	67	4.5MJ/d(-2.5MJ/d)	3	7	4.49	→		→				
152	Hron	2017	CR	F/M	21	60% EI+LF	20	7	13.37	↑		→				
	Hron	2017	CR	F/M		60% EI+LGI	20	7	13.66	↑		→				
	Hron	2017	CR	F/M		60% EI+LC	20	7	13.37	↑		→				
153	Lyngstad	2019	CR	M	40	660kcal/d +keto	8	7	17.20		→	→			↓	→
	Lyngstad	2019	CR	F	55	550kcal/d + keto	8	7	14.96						→	
154	Bowes	2017	CREX	F/M	54	-(400–1000) kcal/d +1h/wk re or aer	32	7	10.54					↓		
155	McNeil	2014	CR	F	65	-633kcal/d	26	7	5.75			→				
155	McNeil	2014	CREX	F	28	-633kcal/d +re	26	7/3	8.22			→				
156	Aukan	2023	CR	F/M	15	750 kcal/d	10	7	14.99		→	→		→		→
157	Triffoni-Melo	2023	CR	F	15	1000 kcal/d + high fibre	0.5	7	2.05	→	→					
	Triffoni-Melo	2023	CR	F	15	1000 kcal/d	0.5	7	2.19	→	→					
158	Purcell	2022	CR	F/M	20.0	-500kcal/d	12	7	3.82	→		→		→		

158	Purcell	2022	EX	F/M	24.0	60%-80% of HRmax	12	4	2.00	→		→		→		
159	Chiurazzi	2023	CR	F/M	27	-40% ER	8	7	3.53	→						↓
160	Åkerström	2022	EX	F	26	27min of HIIT or 40 min of aer	10	3	1.34					→		
161	Mayumi Usuda Prado Rocha	2023	CR	F	15	-500 kcal/d	8	7	2.24		→				↓	
	Mayumi Usuda Prado Rocha	2023	CR	F	14	-500 kcal/d + nuts	8	7	3.87		→				→	
162	Tong 2023	2023	CR	F/M	244	weight-loss diets varying in macronutrient intake	26	7	NA	↑						
163	Hajipoor	2022	CR	F/M	31	regular low fat yogurt + LCD (-500-1000 kcal/d)	10	7	1.03	→		→		→		
164	Edwards	2022	CR	F/M	49	800-880 kcal/d	16	7	4.88	↑					↓	
165	Sommersten	2023	CR	F/M	67	2000 kcal/d(w) and 2500 kcal/d(m) + an acellular high-carbohydrate low-fat (A-HCLF) diet	52	7	5.34	→						
	Sommersten	2023	CR	F/M	62	2000 kcal/d(w) and 2500 kcal/d(m) + a cellular high-carbohydrate low-fat (C-HCLF) diet	52	7	6.20	→						
	Sommersten	2023	CR	F/M	63	2000 kcal/d(w) and 2500 kcal/d(m) + a low-carbohydrate high-fat (LCHF) diet	52	7	7.49	→						
166	Ajami	2022	CREX	F	28	-500-1000kcal/d + low to high intensity exercise + narrative counseling	32	3	16.74	↓						
	Ajami	2022	CREX	F	28	-500-1000kcal/d + low to high intensity exercise + diet and exercise counseling	32	3	10.03	↓						
167	Arjmand	2022	CR	F	22	calorie-restricted MIND diet	12	7	4.86	↑				↑		
	Arjmand	2022	CR	F	15	calorie-restricted waiting list control diet	12	7	2.84	↓				→		
168	Ragland	2023	CR	F	13	low calorie diet	2	7	2.45		→					
	Ragland	2023	CREX	F	12	60min cycling 3min (50% + 90%) HRpeak	2	6	1.42		→					
169	Otten	2019	CR	F	26	Paleolithic diet	104	7	9.59					→		
	Otten	2019	CR	F	23	healthy control diet	104	7	5.65					↑		

n.int: sample size of intervention group; bw.int(%): body weight loss percentage in intervention group; HPLC: high protein low carbhydrate; HPNC: high protein normal carbhydrate; NPLC: normal protein low carbhydrate; NPNC: normal protein normal carbhydrate; EI: energy intake; EE: energy expenditure; (w) and (m): woman and man; ER: energy requirement; HP: high protein; LC: low carbohydrate; LF: low fat; MD: mediterranean diet; VD: vegetatian diet; re: resistance trainning; HR: heart rate; HB: heavy breakfast; HD: heavy dinner; RMR: resting metabolic rate; MICT: moderate-intensity continous training; HIIT: high-intensity interval training; aer: aerobic training; HSF: high satiating food; LSF: low satiating food; Soy_HPWL: soy-based high protein weight loss diet; Meat_HPWL: meat-based high protein weight loss diet; re: resistance training; mod-en: moderate intensity endurance training; mod-re: moderate intensity resistance training; high-en: high intensity endurance training; PA: physical activity; LED: low energy density;HRR: heart rate reserve; keto: ketogenic diet; LGI: low glycaemic diet; MIND diet: Neurodegenerative Delay Diet.
